# Impact of Age and Sex on COVID-19 Severity Assessed From Radiologic and Clinical Findings

**DOI:** 10.3389/fcimb.2021.777070

**Published:** 2022-02-25

**Authors:** Yauhen Statsenko, Fatmah Al Zahmi, Tetiana Habuza, Taleb M. Almansoori, Darya Smetanina, Gillian Lylian Simiyu, Klaus Neidl-Van Gorkom, Milos Ljubisavljevic, Rasha Awawdeh, Hossam Elshekhali, Martin Lee, Nassim Salamin, Ruhina Sajid, Dhanya Kiran, Sanjay Nihalani, Tom Loney, Antony Bedson, Alireza Dehdashtian, Jamal Al Koteesh

**Affiliations:** ^1^College of Medicine and Health Sciences, United Arab Emirates University, Al Ain, United Arab Emirates; ^2^Mediclinic Parkview Hospital, Dubai, United Arab Emirates; ^3^College of Medicine, Mohammed Bin Rashid University of Medicine and Health Sciences, Dubai, United Arab Emirates; ^4^College of Information Technology, United Arab Emirates University, Al Ain, United Arab Emirates; ^5^Radiology Department, Sheikh Shakhbout Medical City, Al Ain, United Arab Emirates; ^6^Radiology Department, Tawam Hospital, Al Ain, United Arab Emirates

**Keywords:** COVID-19, viral pneumonia, age, sex, machine learning, radiomics, risk stratification, severity

## Abstract

**Background:**

Data on the epidemiological characteristics and clinical features of COVID-19 in patients of different ages and sex are limited. Existing studies have mainly focused on the pediatric and elderly population.

**Objective:**

Assess whether age and sex interact with other risk factors to influence the severity of SARS-CoV-2 infection.

**Material and Methods:**

The study sample included all consecutive patients who satisfied the inclusion criteria and who were treated from 24 February to 1 July 2020 in Dubai Mediclinic Parkview (560 cases) and Al Ain Hospital (605 cases), United Arab Emirates. We compared disease severity estimated from the radiological findings among patients of different age groups and sex. To analyze factors associated with an increased risk of severe disease, we conducted uni- and multivariate regression analyses. Specifically, age, sex, laboratory findings, and personal risk factors were used to predict moderate and severe COVID-19 with conventional machine learning methods.

**Results:**

Need for *O*_2_ supplementation was positively correlated with age. Intensive care was required more often for men of all ages (*p <* 0.01). Males were more likely to have at least moderate disease severity (*p* = 0.0083). These findings were aligned with the results of biochemical findings and suggest a direct correlation between older age and male sex with a severe course of the disease. In young males (18–39 years), the percentage of the lung parenchyma covered with consolidation and the density characteristics of lesions were higher than those of other age groups; however, there was no marked sex difference in middle-aged (40–64 years) and older adults (*≥*65 years). From the univariate analysis, the risk of the non-mild COVID-19 was significantly higher (*p <* 0.05) in midlife adults and older adults compared to young adults. The multivariate analysis provided similar findings.

**Conclusion:**

Age and sex were important predictors of disease severity in the set of data typically collected on admission. Sexual dissimilarities reduced with age. Age disparities were more pronounced if studied with the clinical markers of disease severity than with the radiological markers. The impact of sex on the clinical markers was more evident than that of age in our study.

## 1 Introduction

Although there is a wide body of literature on COVID-19, data on the epidemiological characteristics and clinical features of patients of different age and sex are limited. Extensive studies were conducted to cover mainly pediatric (Lu et al., 2020) and elderly populations (Liu et al., 2020a). These studies seldom include the laboratory findings and radiomics of the patients. Commonly, researchers analyze the association of demographic factors and underlying diseases with hospitalization for COVID-19 (Hsu et al., 2020; Sapey et al., 2020; Deeb et al., 2021).

Gaining an insight into the risk factors for non-mild disease is useful for risk stratification and proper management. For this, practitioners should know whether demographics (e.g., age, sex, ethnicity, nationality) are predictors of the severity and outcomes of COVID-19. Research on this issue is challenging as it requires an observational study covering all levels of disease severity. Commonly, the asymptomatic or mildly symptomatic cases remain unstudied as non-severe patients are not admitted to the hospital. This impacts risk assessment because a study cohort is not representative of the entire population in terms of age. The same issue accounts for the disparities in age-specific COVID-19 mortality rates reported in distinct locations (e.g., in China and Korea) (Dudley and Lee, 2020). However, the results obtained in the present study are free of such limitations because the datasets for our study were collected when all the COVID-19 patients verified with reverse transcription-polymerase chain reaction (PCR) were hospitalized and treated in the in-patient clinic at the beginning of the epidemic in the United Arab Emirates (UAE). In this way, we obtained a comprehensive dataset that was representative of the UAE population with regard to age and sex.

### 1.1 Age-Related Features of COVID-19

Age appears to be a strong risk factor for COVID-19 severity and outcomes, as the percentage of immunocompromised people in a population is linked with the age structure of that population. Below is a brief overview of studies on the age-related features of COVID-19.

Children are not as prone to severe forms of COVID-19 compared to adults. This comes from an analysis of SARS-CoV-2 viral load by patient age (Jones et al., 2021). They are underrepresented in study cohorts so that they seem to be less susceptible to the disease (Lee et al., 2020).

There are controversial findings about the group of young adults. The authors assume that reduced compliance with social distancing in young adults may impact the age-specific rate of morbidity and mortality (Dudley and Lee, 2020).

Elderly patients with COVID-19 are more likely to progress to severe disease (Liu et al., 2020a). Reasonably, age-related comorbidities are the leading reason for the increased mortality observed in this age group (Wang et al., 2020; Yang et al., 2020; Zhou et al., 2020). However, physicians should not necessarily extrapolate the age-related tendencies from the population to the individual level. Otherwise, a patient can be considered either high or low risk based on their age rather than on their actual health status which might lead to improper risk assessment, suboptimal resource allocation, and inadequate patient management.

One of the limitations of the previous studies is a focus on midlife adults and elderly people. Exclusion of younger adults means it is not possible to explore the age-related features of COVID-19 across all age groups (Alkhouli et al., 2020; Bertsimas et al., 2020; Covino et al., 2020). Another common limitation in earlier work on age-related aspects of COVID-19 is that authors do not adjust study samples to account for other risk factors which also correlate with age (e.g., diabetes, hypertension, and other background diseases) (Dudley and Lee, 2020; Guan et al., 2020). The outcomes of these studies cannot be generalized because of the limited study samples (Colombi et al., 2020; Hariyanto et al., 2020; Habuza et al., 2021). Finally, it is not clear whether the verification of SARS-CoV-2 infection with nasal and pharyngeal swab is performed properly in all cases with the symptoms and combined with radiological findings suggestive of COVID-19. This can be a source of false positive findings (Luo et al., 2020; Liu et al., 2020a). Another point of concern is that the number of studies on COVID-19 severity in the elderly is disproportionately higher than those on young adults (Costagliola et al., 2021). As such, the impact of age on COVID-19 disease severity has not yet been studied properly.

### 1.2 Sex as a Stratifying Factor

Sex dissimilarity in infectious diseases. A growing body of literature indicates that infectious diseases can affect men and women at a different level. The reasons for these sex differences are related to socioeconomic status, gender inequities, including occupational exposure (Roberts et al., 1998; Tolhurst et al., 2002; Morgan and Klein, 2019), and a sex gap in immune response (Whitacre et al., 1999).

Women are more likely to present with a wide range of specific and non-specific inflammatory (e.g., upper respiratory tract infection, oral and dental conditions) and autoimmune diseases (Schlagenhauf et al., 2010). The immune modulating effect of the sex hormones may underlie these findings (Whitacre et al., 1999).

There is a sex bias in COVID-19, especially at the early stage of the disease (Takahashi et al., 2020). Sex-specific features of the innate and adaptive immune systems (e.g., a higher number of CD4+ T cells, more robust CD8+ T cell cytotoxic activity, and increased B cell production of immunoglobulin) may account for an advantage in the defense against COVID-19 in females. Women are more likely to synthesize higher levels of antibodies against an inactivated influenza vaccine (Peckham et al., 2020). Furthermore, there are sex disparities in physiological responses to viral diseases. The immune system of females has been reported to be twice as strong as that of males (Klein, 2012).

The sex disparity in the efficiency of the immune response correlates with the disease outcomes; i.e., mortality for COVID-19 is twice as common in males (Williamson et al., 2020). Some researchers have associated these findings with genes allocated in the X chromosome (Pontecorvi et al., 2020).

Estrogens may potentiate immune activities of vitamin D, thus improving infection outcomes (Pagano et al., 2020). Conversely, male sex hormones make men vulnerable to COVID-19 and worsen the disease prognosis. First, they are thought to promote viral entry by increasing the activity of the ACE2 receptor—the entry point for the SARS-CoV-2 coronavirus. Second, testosterone exerts immunosuppressive effects and may blunt antibody response. Men may benefit from stimulants of T-cell immune responses and anti-testosterones. Estrogens can be administered to reduce COVID-19 disease severity (Wray and Arrowsmith, 2021).

Researchers aimed to study sex-related differences in COVID-19-associated mortality in multinational cohorts. For this, they used data from national registries or hospitals. Male and female cohorts were not equal in the sets of parameters: age, chronic obstructive pulmonary disease, nicotine dependence, and the total number of comorbidities, including obesity and heart failure (Alkhouli et al., 2020). As the sex groups were not identical, new studies are required to justify a lower mortality from COVID-19 for women and to understand the factors accounting for this sex difference.

Socioeconomic aspects. After adjusting the death rate to socioeconomic factors, authors found that mortality is higher in men in disparate ethnic groups. The relative risk of death from COVID-19 in men compared to women ranges from 1.3 to 3.5 times in different ethnicities (Islam et al., 2020). It remains unknown if socioeconomic inequality accounts for the sex disproportion in the outcomes of COVID-19 (Islam et al., 2020). Reports on the socioeconomic risk factors of COVID-19 are limited. Many studies have focused on the reverse impact of COVID-19 on gender equality (Alon et al., 2020) and the gender gap in work hours (Jin et al., 2020). A study from China showed that men and women have equal chances of getting the disease. However, men with COVID-19 are at a greater risk of worse outcomes and death, independent of age (Jin et al., 2020).

Taking into consideration the results and limitations of recent studies, we aimed to address differential susceptibility of men and women to COVID-19 in order to develop sex-specific intervention strategies. More epidemiological studies should be done throughout the world to compare the burden of the viral disease on human communities in desperate regions (Crighton et al., 2007).

## 2 Objectives

To investigate whether age and sex interact with other risk factors to influence the severity of SARS-CoV-2 infection, we addressed the following objectives:

- Compare the severity of COVID-19 assessed from the radiological findings in patients of different ages and sex.
- Explore the differences between people of distinct age groups and sex with regard to disease severity estimated from the clinical and laboratory findings.
- Measure the informative value of sex and age along with the laboratory findings and personal risk factors for predicting the severity of COVID-19.

## 3 Materials and Methods

### 3.1 Study Design and Sample

The current study analyzed retrospective data obtained as a part of standard primary and secondary care. The study sample included all consecutive patients who satisfied the inclusion criteria (see below section) and who were treated from February 24 to July 01, 2020, in Dubai Mediclinic Parkview (560 cases) or Al Ain Hospital (AAH; 605 cases). The demographic characteristics of both the study cohorts are described in **Table 1**. AAH provides medical care to the second largest city in the Abu Dhabi Emirate. The inclusion criteria were as follows: age 18 years or older; in-patient admission; SARS-CoV-2-positive real-time reverse transcription PCR from nasopharyngeal swabs only, at the hospital site. As mentioned in our previous paper (Statsenko et al., 2021), the novel features of the study are that due to the UAE-wide COVID-19 regulations at the time of the study period all patients with COVID-19 verified by PCR were hospitalized, and we observed all the disease forms (from mild to severe) with a broad spectrum of analyses and radiologic examinations performed. Examinations were conducted in all cases regardless of disease severity. The criteria for assessing disease severity differed by study site (Abu Dhabi versus Dubai) due to the data available at the time of the retrospective study. In the dataset from Al Ain Hospital, the assessment was based on the percentage of the damage to the lungs. Contrarily, in the dataset from Mediclinic Parkview Hospital, the severity level was measured in accordance with the clinical and biochemical signs (see *Methods Used*). The information from electronic health records was summarized using a standardized data collection form adapted from ISARIC Rapid Case Record Form (Harrison et al., 2020).

**Table 1:** Characteristics of study samples.

Study center	Abu Dhabi Emirate Dataset						Dubai Emirate Dataset					
	Al Ain Hospital						Dubai Mediclinic Parkview Hospital					
DEMOGRAPHICS												
	Totaln = 605		Femalen = 86		Malen = 519		Totaln = 560		Femalen = 189		Malen = 371	
- 18–39 yo	341	56.36%	47	54.65%	294	56.65%	292	52.14%	119	62.96%	173	46.63%
- 40–64 yo	253	41.81%	33	38.37%	220	42.39%	236	42.14%	55	29.10%	181	48.79%
- ≥65 yo	11	1.81%	6	6.98%	5	0.96%	32	5.71%	15	7.94%	17	4.58%
SEVERITY OF COVID-19												
Asymptomatic/mild	357	59.01%	54	62.79%	303	58.38%	343	61.25%	129	68.25%	214	57.68%
Moderate	215	35.54%	28	32.56%	187	36.03%	88	15.71%	29	15.34%	59	15.9%
Severe	31	5.12%	4	4.65%	27	5.20%	83	14.82%	20	10.58%	63	16.98%
Critical	2	0.33%	0	0.00%	2	0.39%	46	8.21%	11	5.82%	35	9.43%
Criteria of severity level	Radiological criteria						Clinical criteria					
*- Mild form*	Lung involvement *<* 5% total lung volume						Clinical symptoms of upper respiratory tract infection and no signs of pneumonia					
*- Moderate form*	Lung involvement [5%, 25%) total lung volume						Fever and respiratory symptoms with radiological findings of pneumonia					
*- Severe form*	Lung involvement [25%, 50%) total lung volume						ARDS: respiratory rate > 30/min, SpO2 <93% at rest, P/F ratio <300					
*- Critical form*	Lung involvement *≥* 50% total lung volume						Any of the following: P/F ratio < 200, sepsis, multiorgan failure, GCS<13					

### 3.2 Patient and Public Involvement

There was no patient involvement as the data were collected retrospectively from the medical record system and PACS server.

### 3.3 Methods Used

*To address the first objective*, we divided the study samples into groups and used descriptive statistics. The range of years corresponding to the age groups was 18–39 years for young adults, 40–64 for midlife adults, and 65 years and over for older adults.

As the variables of the datasets were distributed non-normally, we utilized non-parametric tests for the analysis. In the age groups, the relationships between the continuous features were assessed with the Kruskal–Wallis test. With this test, we also examined the sex-related differences within each age group.

This part of our study was conducted with the dataset from AAH. We used radiomics to estimate disease severity as, evidently, the level of the lung involvement in CT correlates with disease severity. In analogy to the existing scoring systems (e.g., lung CT score), we applied the following thresholds: mild cases had <5%, moderate *∈* [5, 25), severe *∈* [25, 50), and critical ones *≥*50% lung involvement.

To collect the radiomics data for the entire lung and their lobes, we applied lung masks. The masks were segmented with the deep learning U-net model trained on a large and diverse dataset (Hofmanninger et al., 2020). Ground glass opacity (GGO), consolidation, and pleural effusion are the most common types of the lung lesions in COVID-19. These lesions were segmented with the CT Thorax COVID-19 model from MedSeg tool (COVID-19 CT Segmentation Dataset, 2021). By multiplying the number of voxels in the mask by the voxel size, we received the total lung volume as well as the volumes of the lung lobes. We also segmented pathology lesions. To calculate the mean density, its standard deviation, and entropy, we utilized the fslstats tool from the FSL framework (Jenkinson et al., 2012). The characteristics of density were in Hounsfield units (HU). Finally, all volume variables were normalized or expressed as a percentage to the total lung volume. To study the association of the radiomical features, clinical signs, laboratory findings, comorbidities, and complications with age, sex, and disease severity, we calculated Pearson’s correlation coefficients and assessed their significance.

*To address the second objective*, we used the same methods of descriptive statistics as in the first objective but with a different dataset. This allowed us to verify the findings. In the Dubai Mediclinic Parkview Hospital sample, disease severity was determined according to the National Guidelines National Emergency Crisis and Disasters Management Authority (2020) in the following way: *an asymptomatic form* referred to a patient with no symptoms; *a mild form*—clinical symptoms of upper respiratory tract infection and no signs of pneumonia; *a moderate form*—fever and respiratory symptoms with radiological findings of pneumonia; *a severe form*—any of the following criteria: respiratory distress (RR > 30/min), oxygen saturation <93% at rest, P/F ratio of less than 300; and *a critical form* fitted any of the following criteria: ARDS (P/F ratio < 200), sepsis, multiorgan failure, and altered level of consciousness (GCS < 13).

We followed the same procedure as in the first objective to assess the association between the clinical and laboratory findings in both datasets by computing Pearson’s correlation coefficients.

*To achieve the third objective*, we used a set of statistical approaches. First, we used univariate and multivariate analyses to investigate which factors were associated with the non-mild form of COVID-19 (see **Table 2**). This allowed us to calculate the adjusted odds ratio associated with age and sex used either as single predictors or in combination with the data typically collected on admission, i.e., the results of the physical examination (body mass index, blood pressure, body temperature, heart rate, breath rate) and hypoxia markers (SpO2, HCO3, serum potassium, anion gap).

**Table 2:** Univariate and multivariate analyses of age and sex factors on severity of the disease (severity is at least moderate).

Variable	Severity assessed radiologically, Abu Dhabi Emirate Dataset			Severity assessed clinically, Dubai Emirate Dataset		
	aOR	95% CI	p	aOR	95% CI	p
Univariate analysis						
Age	1.028	[1.014–1.044]	**<0.001**	1.08	[1.06–1.10]	**<0.001**
Young adults, 18*−*39 yo	Ref.	–	–	Ref.	–	–
Midlife adults, 40*−*64 yo	1.52475	[1.09–2.12]	**0.01265**	5.55	[3.47–9.14]	**<0.001**
Older adults, *≥* 65 yo	4.72629	[1.34–21.88]	**0.02364**	17.05	[7.62–39.83]	**<0.001**
Females	Ref.	–	–	Ref.	–	–
Males	1.2	[0.76–1.94]	0.44	1.82	[1.18–2.9]	**0.0083**
Multivariate analysis						
Age	1.02	[1.00–1.03]	*0.06869*	1.07	[1.05–1.10]	**2.55e-09**
Sex	1.36	[0.81–2.32]	0.25640	1.93	[1.02–3.82]	*0.050473*
BMI	1.01	[0.99–1.02]	0.31163	1.11	[1.03–1.19]	**0.003459**
SpO_2_	0.00	[0.00–0.85]	*0.06421*	0.61	[0.53–0.70]	**8.49e-11**
Systolic blood pressure	1.00	[0.99–1.02]	0.43913	1.00	[0.98–1.02]	0.95765
Diastolic blood pressure	0.98	[0.97–1.00]	**0.01982**	0.99	[0.95–1.02]	0.365582
Heart rate	1.01	[1.00–1.03]	**0.03488**	1.01	[0.99–1.04]	0.246975
Temperature	1.33	[0.83–2.14]	0.23986	2.62	[1.79–3.91]	**1.25e-06**
Breath rate	1.10	[0.97–1.26]	0.15006			
HCO3	0.93	[0.84–1.02]	0.12393			
Serum potassium	1.34	[0.82–2.18]	0.24068			
Anion gap	0.96	[0.87–1.07]	0.46646			

aOR, adjusted odds ratio; BMI, body mass index; CI, confidence interval; SpO2, oxygen saturation.

Significant associations between variables and disease severity (p < 0.05) are marked in bold font.

Then, we employed machine learning (ML) classification models. To start with, all the informative features from AAH were ranked by their potential to predict disease severity. To assess the importance of the features fed into the ML models as predictors, we employed four ensemble tree-based estimators such as AdaBoost, Gradient Boosting, Random Forest, and Extra Trees. These models were trained on the whole dataset and used to rank the features in ascending order concerning their predictive potential. As the dataset was unbalanced due to common reasons (severe and critical disease forms are less common than the mild ones), we built a binary classification to distinguish the mild disease from the other forms (357 vs. 248 cases in AAH; 431 vs. 129 in Dubai). To test the impact of age and sex on the disease course, we utilized different sets of predictors. Particularly, we merged the data on age and sex with the laboratory findings from the AAH dataset. In the Dubai Mediclinic dataset, we selected personal risk factors, including background diseases, age, and sex, to show the most informative features. To provide further evidence of the importance of age and sex as predictors of COVID-19 severity, we compared the accuracy of machine learning models built with and without them (see **Table 3**).

**Table 3:** Performance of classification models predicting severity of COVID-19 from laboratory findings (Abu Dhabi dataset) or individual risk factors (Dubai dataset) with and without age and sex.

ML method		Prediction of severity from					Prediction of disease severity from				
		laboratory findings, age and sex					individual risk factors inclusively age and sex				
		Precision	Recall	F1	AUC	Acc	Precision	Recall	F1	AUC	Acc
AdaBoost	w/o	0.63	0.64	0.62	0.6444	0.64	0.72	0.73	0.72	0.641	0.73
	with	0.64	0.64	0.63	0.6585	0.64	0.72	0.74	0.73	0.7222	0.74
ExtraTrees	w/o	0.68	0.68	0.65	0.6658	0.68	0.73	0.76	0.74	0.6824	0.76
	with	0.71	0.69	0.67	0.6728	0.69	0.74	0.76	0.75	0.7452	0.76
Random forest	w/o	0.72	0.69	0.66	0.6633	0.69	0.73	0.78	0.73	0.709	0.78
	with	0.72	0.69	0.66	0.6887	0.69	0.74	0.78	0.73	0.7998	0.78
NN	w/o	0.70	0.71	0.70	0.7547	0.71	0.76	0.79	0.76	0.7208	0.79
	with	0.79	0.77	0.76	0.7906	0.77	0.80	0.81	0.80	0.8134	0.81
SVM (linear)	w/o	0.66	0.66	0.63	0.6465	0.66	0.68	0.76	0.69	0.6656	0.76
	with	0.67	0.67	0.64	0.6534	0.67	0.71	0.76	0.71	0.7806	0.76
LR	w/o	0.65	0.66	0.64	0.655	0.66	0.74	0.78	0.74	0.7239	0.78
	with	0.66	0.66	0.65	0.6643	0.66	0.74	0.78	0.74	0.7863	0.78
**Gain after adding age and sex to predictors**		+3%	+1.33%	+1.83%	+1.64%	+1.33%	+1.5%	+0.5%	+1.33%	**+8.41%**	+0.5%

Significant differences (p < 0.05) between models performance with and without such predictors as age and sex are marked in bold font.

AUC, area under the receiver operating characteristic curve; Acc, accuracy; LR, logistic regression; ML, machine learning; SVM, support vector machine; w/o, without.

We used the computational power of the Linux Ubuntu 18.04 NVIDIA DGX-1 deep learning server with 40 CPU cores and 8x NVIDIA Tesla V100 GPU with 32 GB memory each, accessed with a web-based multiuser concurrent job scheduling system (Habuza et al., 2020). The experimental work was conducted using programming languages R, Python, and its libraries for deep learning, data processing, and data visualization, such as tensorflow-gpu, keras, Sklearn, SciPy, NumPy, Pandas, Matplotlib, and Seaborn.

## 4 Results

### 4.1 Sex and Age Disparities in Disease Severity Assessed From Radiomics

As expected, the lung volume was smaller in women and in older adults (see **Table 4**). The interpretation of age- and sex-specific differences in some radiomics data (e.g., lungs entropy, density) was challenging because of an unclear clinical value of the data. In young and middle-aged adults, there was a marked sex disproportion in the location of the center of gravity along the axial axis. This may have resulted from a more severe lung involvement in men and up–down gradient in the distribution of the lung lesions in COVID-19-associated pneumonia.

**Table 4:** Radiomics data on lung involvement f subjects with regard to age groups and gender, Abu Dhabi Emirate.

	Total	Both sexes				All ages			18-39 years			40-64 years			*≥*65 years		
		18-39n_1_=341(56.36%)	40-64n_2_=253(41.82%)	*≥*65n_3_=11(1.82%)	p_1−3_	Femalen_4_=86(14.21%)	Malen_5_=519(85.79%)	p_4-5_	Femalen_6_=47(13.78%)	Malen_7_=294(86.22%)	p_6-7_	Femalen_8_=33(13.04%)	Malen_9_=220(86.96%)	p_8-9_	Femalen_10_=6(54.55%)	Malen_11_=5(45.45%)	p_10-11_
**GENERAL LUNG RADIOMICS**																	
Lung volume, *L*	3.4[2.62-3.96]	3.44 ± 1.08	3.37 ± 0.99	3.02 ± 1.24	0.2157	2.68 ± 0.82	3.52 ± 1.03	**<0.001**	2.82 ± 0.93	3.54 ± 1.07	**<0.001**	2.57 ± 0.66	3.49 ± 0.97	**<0.001**	2.21 ± 0.3	3.98 ± 1.25	**0.0179**
Lung entropy	0.71[0.69-0.74]	0.71 ± 0.03	0.71 ± 0.04	0.74 ± 0.06^*^	0.0636	0.71 ± 0.05	0.71 ± 0.04	0.2814	0.71 ± 0.03	0.71 ± 0.03	0.4965	0.7 ± 0.05	0.71 ± 0.04	0.171	0.72 ± 0.07	0.78 ± 0.04	0.1177
**Density**																	
- maximal	664.27[547.0-700.0]	645.37 ± 236.11^*^	692.11 ± 234.5^*^	610.0 ± 126.04	**0.0013**	657.86 ± 192.45	665.34 ± 241.46	0.2057	626.0 ± 143.67	648.47 ± 247.57	0.3197	706.42 ± 244.93	689.96 ± 232.82	0.196	640.33 ± 134.02	573.6 ± 104.71	0.324
- mean	-70.16[-85.06–52.14]	-73.42 ± 25.46^*^	-65.88 ± 22.34^*^	-67.22 ± 27.64	**0.0024**	-68.46 ± 24.31	-70.44 ± 24.56	0.2273	-75.43 ± 24.4	-73.1 ± 25.61	0.2369	-60.36 ± 20.43	-66.71 ± 22.49	0.0597	-58.41 ± 25.88	-77.8 ± 25.93	0.0603
- std deviation	221.02[190.24-250.95]	226.56 ± 41.97^*^	214.02 ± 40.32^*^	210.11 ± 47.36	**0.0033**	217.02 ± 42.4	221.68 ± 41.75	0.1633	229.23 ± 40.59	226.14 ± 42.17	0.3064	203.87 ± 38.57	215.54 ± 40.35	0.053	193.61 ± 44.89	229.9 ± 42.4	0.0855
**Center of gravity**																	
- lateral axis (x)	255.71[255.24-256.09]	255.74 ± 0.86	255.66 ± 0.72	255.75 ± 0.82	0.8183	255.76 ± 0.82	255.7 ± 0.8	0.232	255.79 ± 0.87	255.73 ± 0.85	0.3087	255.71 ± 0.7	255.66 ± 0.72	0.2689	255.8 ± 1.01	255.69 ± 0.51	0.4636
- anterior-posterior axis (y)	256.68[255.49-257.7]	256.78 ± 2.03	256.52 ± 1.75	257.1 ± 1.56	0.1201	257.33 ± 1.78	256.57 ± 1.91	**<0.001**	257.8 ± 1.79	256.62 ± 2.01	**<0.001**	256.6 ± 1.59	256.51 ± 1.77	0.1877	257.74 ± 1.33	256.33 ± 1.45	0.0855
- axial axis (z)	154.62[143.66-165.03]	153.89 ± 15.27	156.06 ± 15.77	144.33 ± 15.91^*^	**0.0398**	145.26 ± 14.67	156.17 ± 15.19	**<0.001**	145.13 ± 14.76	155.29 ± 14.88	**<0.001**	146.72 ± 15.06	157.46 ± 15.39	**0.0002**	138.19 ± 8.38	151.71 ± 19.3	0.0855
**SEVERITY LEVEL ASSESSED FROM LUNG INVOLVEMENT**																	
**Severity level**																	
- mild	357(59.01%)	218(63.93%)^*^	136(53.75%)^*^	3(27.27%)	**0.0005**	54(62.79%)	303(58.38%)	0.833	34(72.34%)	184(62.59%)	0.2598	18(54.55%)	118 (53.64%)	0.9075	2 (33.33%)	1 (20.0%)	
- moderate	215(35.54%)	113(33.14%)	97(38.34%)	5(45.45%)		28(32.56%)	187(36.03%)		13(27.66%)	100(34.01%)		12(36.36%)	85 (38.64%)		3 (50.0%)	2 (40.0%)	
- severe	31(5.12%)	10(2.93%)^*^	18(7.11%)	3(27.27%)*		4(4.65%)	27(5.2%)		0(0.0%)	10(3.4%)		3(9.09%)	15 (6.82%)		1 (16.67%)	2 (40.0%)	
- critical	2(0.33%)	0(0.0%)	2(0.79%)	0(0.0%)		0(0.0%)	2(0.39%)					0(0.0%)	2 (0.91%)				
**LUNG INVOLVEMENT**																	
**Involvement**, %	6.71[0.81-9.24]	5.07 ± 6.79^*^	8.55 ± 10.58^*^	15.36 ± 11.95^*^	**<0.001**	6.7 ± 7.93	6.71 ± 9.09	0.3984	3.79 ± 4.24	5.27 ± 7.1	0.1181	9.47 ± 9.28	8.42 ± 10.76	0.1406	14.28 ± 10.98	16.66 ± 12.9	0.3921
Emphysema, %	0.65[0.06-0.59]	0.68 ± 1.5	0.61 ± 1.17	0.6 ± 1.16	0.9977	0.43 ± 0.9	0.69 ± 1.43	**0.0021**	0.53 ± 1.13	0.71 ± 1.55	0.0534	0.32 ± 0.52	0.65 ± 1.24	**0.0126**	0.22 ± 0.23	1.07 ± 1.58	0.1577
GGO, %	5.43[0.45-7.87]	4.13 ± 5.77^*^	6.83 ± 8.28^*^	13.71 ± 10.68^*^	**<0.001**	5.88 ± 7.19	5.35 ± 7.25	0.1682	3.41 ± 4.05	4.24 ± 5.99	0.3505	8.07 ± 8.28	6.64 ± 8.27	0.0979	13.19 ± 10.42	14.34 ± 10.94	0.4636
- max density, *HU*	11.05[-0.0–0.0]	7.03 ± 20.54^*^	15.92 ± 29.08^*^	23.82 ± 29.35^*^	**<0.001**	5.31 ± 14.64	12.0 ± 26.29	**0.0225**	1.23 ± 8.37	7.95 ± 21.72	**0.0061**	10.18 ± 19.64	16.78 ± 30.15	0.168	10.5 ± 10.56	39.8 ± 35.96	0.1461
- mean density, *HU*	-2.4[-3.67–0.26]	-1.9 ± 2.16^*^	-2.9 ± 3.2^*^	-6.27 ± 4.95^*^	**<0.001**	-2.59 ± 2.83	-2.37 ± 2.8	0.1970	-1.76 ± 1.93	-1.92 ± 2.19	0.3720	-3.26 ± 3.04	-2.85 ± 3.22	0.1434	-5.36 ± 4.38	-7.37 ± 5.35	0.3921
- std density, *HU*	32.07[13.38-47.59]	28.88 ± 18.82^*^	35.4 ± 22.45^*^	54.04 ± 24.47^*^	**0.0001**	33.7 ± 21.06	31.8 ± 20.96	0.2202	27.74 ± 18.37	29.06 ± 18.88	0.3496	39.02 ± 21.59	34.86 ± 22.53	0.1523	51.1 ± 20.38	57.58 ± 28.21	0.3921
Consolidation, %	1.28[0.14-1.08]	0.94 ± 1.97^*^	1.73 ± 3.74^*^	1.65 ± 1.78	**<0.001**	0.82 ± 1.43	1.36 ± 3.04	**0.0016**	0.38 ± 0.59	1.03 ± 2.09	**<0.001**	1.4 ± 2.01	1.78 ± 3.93	0.3827	1.09 ± 0.89	2.31 ± 2.28	0.324
- max density, *HU*	145.31[89.0-174.0]	126.05 ± 105.04^*^	169.0 ± 158.88^*^	197.55 ± 96.18^*^	**<0.001**	112.51 ± 105.53	150.74 ± 135.18	**0.0005**	79.91 ± 62.2	133.42 ± 108.56	**<0.001**	149.12 ± 140.93	171.98 ± 161.19	0.1792	166.5 ± 38.43	234.8 ± 126.63	0.2614
- mean density, *HU*	-0.15[-0.09–0.01]	-0.08 ± 0.19^*^	-0.24 ± 0.62^*^	-0.3 ± 0.38^*^	**<0.001**	-0.08 ± 0.18	-0.16 ± 0.46	**0.0289**	-0.03 ± 0.06	-0.09 ± 0.21	**0.0022**	-0.14 ± 0.26	-0.25 ± 0.65	0.3827	-0.16 ± 0.2	-0.46 ± 0.47	0.1177
- std deviation, *HU*	4.89[1.9-5.82]	3.76 ± 3.33^*^	6.28 ± 6.19^*^	7.97 ± 4.84^*^	**<0.001**	3.77 ± 3.21	5.08 ± 5.14	**0.0092**	2.52 ± 1.85	3.96 ± 3.47	**0.0008**	5.13 ± 3.83	6.45 ± 6.45	0.2689	5.97 ± 3.58	10.37 ± 5.05	0.1177
**SPECIFIC LOBE INVOLVEMENT**																	
*Left upper lobe*																	
- GGO, %	1.73[0.15-2.47]	1.44 ± 2.13^*^	2.04 ± 2.62^*^	3.5 ± 3.21^*^	**0.0041**	2.06 ± 2.51	1.68 ± 2.38	**0.0221**	1.36 ± 1.58	1.45 ± 2.21	0.1985	2.74 ± 3.08	1.94 ± 2.52	0.0556	3.68 ± 3.08	3.29 ± 3.34	0.4636
- consolidation, %	0.17[0.01-0.11]	0.1 ± 0.27^*^	0.26 ± 0.54^*^	0.24 ± 0.3^*^	**<0.001**	0.11 ± 0.23	0.18 ± 0.44	**0.0249**	0.03 ± 0.08	0.12 ± 0.29	**0.0011**	0.2 ± 0.29	0.26 ± 0.57	0.4614	0.23 ± 0.33	0.25 ± 0.26	0.4636
*Left lower lobe*																	
- GGO, %	0.45[0.01-0.25]	0.24 ± 0.81^*^	0.66 ± 1.37^*^	2.14 ± 2.14^*^	**<0.001**	0.5 ± 1.02	0.44 ± 1.17	0.1373	0.15 ± 0.38	0.25 ± 0.85	0.3118	0.75 ± 1.1	0.65 ± 1.4	0.0795	1.89 ± 1.96	2.44 ± 2.3	0.4636
- consolidation, %	0.09[0.0-0.03]	0.04 ± 0.18^*^	0.15 ± 0.43^*^	0.11 ± 0.14^*^	**<0.001**	0.07 ± 0.23	0.09 ± 0.33	0.1883	0.01 ± 0.01	0.04 ± 0.19	**0.0034**	0.17 ± 0.34	0.14 ± 0.44	0.1602	0.08 ± 0.1	0.14 ± 0.16	0.3921
*Right upper lobe*																	
- GGO, %	0.51[0.01-0.38]	0.26 ± 0.63^*^	0.77 ± 1.59^*^	2.41 ± 2.94^*^	**<0.001**	0.49 ± 1.06	0.52 ± 1.28	0.381	0.13 ± 0.17	0.28 ± 0.68	0.1725	0.69 ± 1.01	0.78 ± 1.66	0.2317	2.22 ± 2.47	2.64 ± 3.41	0.4636
- consolidation, %	0.07[0.0-0.02]	0.03 ± 0.2^*^	0.12 ± 0.4^*^	0.08 ± 0.07^*^	**<0.001**	0.03 ± 0.07	0.08 ± 0.32	0.1703	0.0 ± 0.01	0.04 ± 0.22	**0.0172**	0.06 ± 0.09	0.13 ± 0.42	0.4584	0.07 ± 0.06	0.08 ± 0.07	0.4636
*Right middle lobe*																	
- GGO, %	0.11[0.0-0.04]	0.05 ± 0.18^*^	0.17 ± 0.41^*^	0.63 ± 0.73^*^	**<0.001**	0.11 ± 0.35	0.11 ± 0.32	0.222	0.02 ± 0.04	0.05 ± 0.19	0.4297	0.18 ± 0.43	0.17 ± 0.41	0.1828	0.5 ± 0.68	0.79 ± 0.77	0.2614
- consolidation, %	0.02[0.0-0.01]	0.01 ± 0.03^*^	0.04 ± 0.15^*^	0.04 ± 0.05	**<0.001**	0.02 ± 0.08	0.02 ± 0.1	0.0849	0.0 ± 0.0	0.01 ± 0.04	**0.0287**	0.04 ± 0.12	0.04 ± 0.15	0.4237	0.05 ± 0.07	0.03 ± 0.03	**0.5**
*Right lower lobe*																	
- GGO, %	2.48[0.13-4.0]	2.05 ± 2.62^*^	2.99 ± 3.29^*^	4.43 ± 2.8^*^	**0.0001**	2.57 ± 3.11	2.47 ± 2.95	0.4147	1.66 ± 2.24	2.11 ± 2.67	0.1583	3.52 ± 3.68	2.91 ± 3.22	0.1762	4.52 ± 3.03	4.32 ± 2.48	0.4636
- consolidation, %	0.17[0.0-0.1]	0.1 ± 0.36^*^	0.27 ± 0.64^*^	0.2 ± 0.17^*^	**<0.001**	0.1 ± 0.27	0.19 ± 0.53	0.0825	0.02 ± 0.05	0.12 ± 0.39	**0.0099**	0.19 ± 0.4	0.28 ± 0.67	0.4316	0.12 ± 0.1	0.29 ± 0.19	0.0603

a Statistical data are expressed as IQR, Median ± SD, or the absolute number of cases and their percentage in the sample studied.

If the distribution of a variable differs significantly (p <0.05) in an age group plotted against all the other ones, its value is marked with an asterisk.

Disparities in the distribution of data across age and sex groups are presented with p-values in separate columns. The p-value is marked in bold if the difference between the groups is statistically significant (p < 0.05).

The portion of mild cases decreased dramatically with age. The severity of COVID-19 assessed from the percentage of lung involvement did not differ between sexes. The distribution of the lung lesions over specific lobes was common for distinct age groups and sexes.

Men had a greater lung volume compared to women (p*≤* 0.0179). The portion of the lung with emphysematous changes was larger in men. Sex disparity in the lung involvement was significant in young adults: there were differences in the density characteristics of ground glass opacity (GGO) and consolidation. The data reflect a more severe lung involvement in men; i.e., the lesions were denser in males compared to their counterparts of the same age (*p <* 0.05). The total involvement of the left lower lobe, right middle lobe, and right lower lobe and the percentage of the lung parenchyma covered with consolidation were higher in men. In older adults, there were no marked differences between sexes. In midlife adults, such differences were also minimal with the exception of a statistically larger center of gravity along the axial axis in males (*p* = 0.0002). The results suggest that dissimilarities in the hormonal status may underlie differences between sexes in COVID-19. As the level of hormonal activity reduces with age, the sex disparities in the disease also decreases.

**Figure 1:**
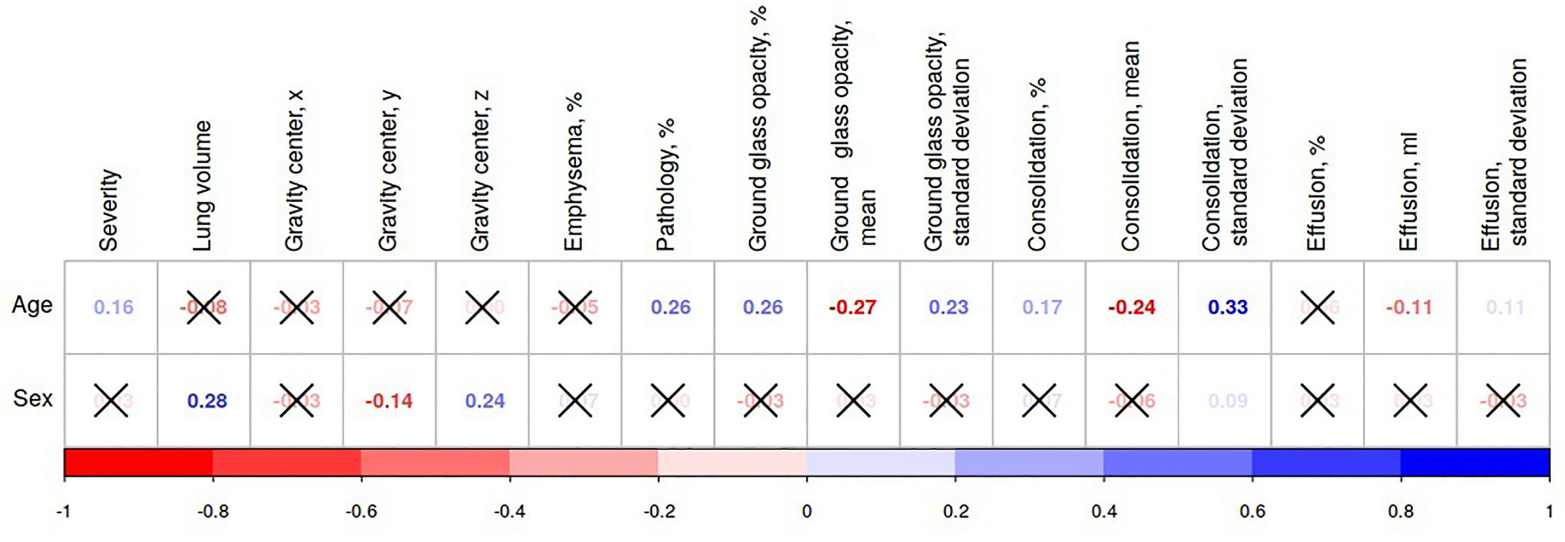
Association of radiomical features with age and sex in the datasets from the Emirate of Abu Dhabi. If an association between variables is significant (*p <* 0.05), the values of Pearson’s correlation coefficients for it are presented in the diagram; otherwise, the values are crossed out.

Pearson’s correlation coefficients show the relationship between age, sex, and radiomical findings (see **Figure 1**). The level of severity assessed with the percentage of the lung involvement had a strong positive correlation with age (*r* = 0.16, *p* = 0.0001). However, there was no statistical association between the severity marker and sex (*r* = 0.003, *p* = 0.44).

### 4.2 Differences Between Sex and Age Groups in COVID-19 Severity as per Clinical Data and Laboratory Findings

#### 4.2.1 Laboratory Findings

From our data, the majority of findings were within the reference norm as the mild form of COVID-19 was most prevalent in the study cohort (see **Supplemental Tables 1, 2**) The biochemical findings in both datasets support the relationships identified from radiomics and briefly described in *Sex and Age Disparities in Disease Severity Assessed From Rediomics*. The statistical analysis of the laboratory data supports the trend toward worsening of the SARS-CoV-2 infection with age. Sexual dissimilarities were also pronounced at the age of active hormonal changes and decreased with age.

The analysis of the coagulatory system and biochemical markers illustrated the common observations in both study cohorts. The level of CRP is an important marker of disease severity, and it showed the same association with advancing age as the radiological markers of lung involvement. The majority of substrates demonstrate a greater count in men than in women, although a significant difference (*p <* 0.05) was observed only for a few laboratory findings. This suggests that, in general, men appear to suffer more severe cases and die of the disease at greater rates. Older patients are more likely to have hypercoagulation and clot formation as they have a higher level of APTT, D-Dimer, and fibrinogen compared to the younger groups (*p <* 0.001). Moreover, the level of D-dimer deviated from the clinical reference values (*>*0.5 ug/l) only in older adults.

An increase in lactate dehydrogenase (LDH) and creatine kinase (CK) activity may reflect energy deficiency caused by hypoxia. The levels of these enzymes were lower in young adults than in midlife or older-aged patients (p *≤* 0.003). Although there was a marked difference in the level of alanine aminotransferase (ALT) and aspartate aminotransferase (AST) between the three age groups, it was not possible to confirm a direct association between the elevated levels of these enzymes with age as midlife adults had the highest enzyme activity. The Dubai-based cohort demonstrated a profound difference in the enzyme level between sexes and between midlife and young adults (*p <* 0.001). The variance of the platelet, white blood cell (WBC), and red blood cell (RBC) count among patients of distinct age was not clinically relevant.

**Figure 2:**
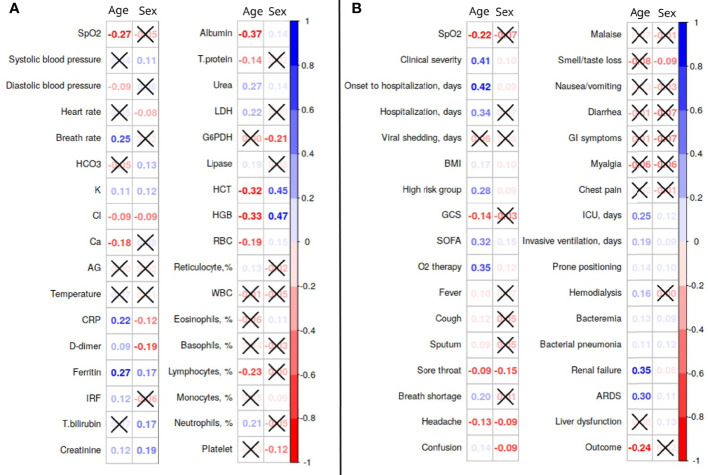
Association of clinical and laboratory features with age and sex in the datasets from the Emirate of Abu Dhabi **(A)** and Dubai **(B)**. AG, anion gap; ARDS, acute respiratory distress syndrome; BMI, body mass index; CRP, C-reactive protein; G6PDH, glucose-6-phosphate dehydrogenase; GCS, Glasgow Coma Scale; GI, gastrointestinal; HGB, hemoglobin; HCT, hematocrit; ICU, intensive care unit; IRF, immature reticulocyte fraction; LDH, lactate dehydrogenase; RBC, red blood cells; SpO2, oxygen saturation; SOFA, sequential organ failure assessment; T.bilirubin, total bilirubin; T.protein, total protein; WBC, white blood cells.

These were associations between age, sex, and the major clinical parameters and laboratory findings studied in two clinics. The markers of the disease progression indicate worsening of the disease course with advancing age. This was true for the numerical values of the laboratory findings (see **Figure 2A**) and appearance of the clinical symptoms (see **Figure 2B**). The level of clinical severity collected in Dubai dataset expressed a moderate-strength positive correlation with age (*r* = 0.41, *p <* 0.0001).

From the same data, there was a very weak association of male sex with severe disease (*r* = 0.10, *p* = 0.13).

#### 4.2.2 Clinical Findings, Patient Management, and Outcomes

In the dataset from Dubai, it was possible to assess the severity of COVID-19 directly from the clinical data (*“Clinical severity”*) and indirectly from *“Onset of hospitalization days*”, *“Duration of viral shading*”, *“Disease outcomes*”, symptoms, etc. (see **Supplemental Table 2**). The study data shows that in the age range from 18 to 39 years the disease was more severe in males (*p* = 0.328), but there was no evidence on sex dissimilarities from the indirect markers (*p >* 0.05).

**Figure 3:**
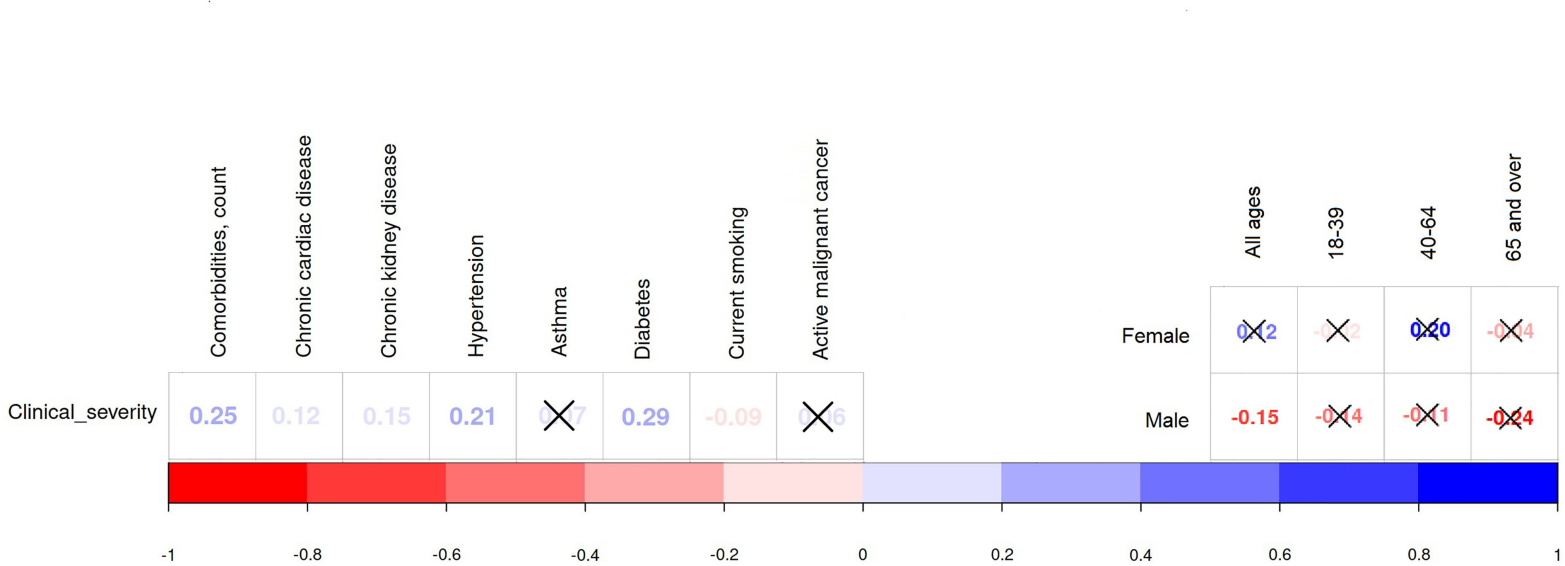
Association of clinical severity with comorbidities (Dubai dataset).

Hypertension was the prevailing comorbidity (20.54%) in the study sample. There was no pronounced sex difference in the prevalence of individuals suffering from hypertension and diabetes mellitus. Comparison of young and older adults shows that more midlife subjects were classified in a severe or critical condition. Disease severity was positively associated with the following background diseases: diabetes mellitus (*r* = 0.29, *p* <0.0001), hypertension (*r* = 0.21, *p* <0.0001), chronic kidney (*r* = 0.15, *p* = 0.0003), and cardiac disease (*r* = 0.12, *p* = 0.0041; see **Figure 3** and **Supplemental Table 3**). There was a weak negative association between current smoking and the severity of COVID-19 (*r* = *−*0.09, *p* = 0.043). This might explain the fact that smoking was twice as common in young adults as in midlife adults (8.22% vs. 4.24%) and there was no marked correlation between current smoking and COVID-19 severity inside age groups (*p >* 0.05).

Male subjects in the age group of 18–39 years had a statistically higher mean BMI. Noticeably, while the BMI of male patients was consistent throughout age groups, the BMI of women increased with age. For instance, in the age bracket of 65 years and over, the BMI was higher than the BMI of males*, p* = 0.0179. Young males had a higher body temperature (°C) than their female counterparts (37.0 *±* 0.67 vs 37.0 *±* 0.47; *p <* 0.0211). Both systolic and diastolic blood pressures were significantly higher in adult males with the p-value varying from less than 0.0001 to 0.0179.

The respiratory rate was slightly higher in males, except for older adults where female patients displayed higher values of breaths per minute (22.0 *±* 6.29 vs. 18.0 *±* 2.22; *p <* 0.0129). The same relationship was observed for SOFA score; specifically, SOFA score was higher in male patients (0.30 *±* 0.94 for young adults and 1.28 *±* 2.32 for midlife adults) than in females (0.04 *±* 0.20 and 0.42 *±* 1.15, respectively) with the p value ranging from 0.0016 to 0.0046. Although they are statistically significant, these differences are hard to interpret in all age groups because they have no notable clinical value.

There was no large difference in the number of comorbidities in any study cohorts, but some were more prevalent in certain age groups. We also analyzed statistics on smoking which is a risk factor for background diseases worsening COVID-19 outcomes (e.g., lung and cardiovascular pathology). Thus, male subjects in the age group of 18–39 years showed a higher percentage of current smokers (12.72 vs. 1.68%; *p <* 0.0004). This trend was not observed in the 40–64-year age group with any significant difference; however, older-aged female subjects had a higher prevalence of asthma compared to men (10.91 vs. 3.31%; *p <* 0.0357). Marked differences between sexes were apparent in chronic cardiac disease. Its prevalence in male patients was much higher than females (13.33 vs. 58.82%; *p <* 0.0118).

Out of 560 patients analyzed, the majority (76.96%) of patients had either an asymptomatic or mild disease form, followed by the severe cases (14.82%), and the fewest patients were in critical condition (8.21%). Young adults were either asymptomatic or had a mild disease form in 90.75% of cases, midlife adults in 65.25%, and older adults only in 37.5% of cases. Only 6.85% of young adults had a severe case of the disease, while 21.61% of midlife and 37.5% of older adults were classified as severe cases. Out of all age groups, older adults had the highest portion of critical cases (25%), followed by midlife adults (13.14%) and young adults (2.4%). 0_2_ supplementation was required in 14.64% of cases, and it was most frequently administered to older adults both on admission (43.75%) and later in the clinics (53.12%). The need for O_2_ supplementation was directly correlated with age. Male patients of all age groups were considerably more likely to require O_2_ supplementation with the p-value from 0.0034 to 0.0257. Among all patients, 56 (10%) were admitted to the ICU directly, and 72 (12.86%) were transferred there later when their condition deteriorated. Older adults were more likely to be admitted to the ICU than midlife and young adults (*p <* 0.001). Male patients were admitted to the ICU more frequently than female patients were (*p <* 0.0027). The time between the onset of the disease and hospitalization as well as between the onset and positive PCR was the longest for older adults followed by midlife and then young adults (*p <* 0.001). The opposite tendency was observed for young adults (*p <* 0.0102). There was evidence of a correlation between age and the duration of the disease (*p <* 0.0009). Male patients had a longer duration of disease in the 40–64- (*p <* 0.0259) and above 65-year-old (*p <* 0.0467) age groups. The outcome of the disease was reported to be fatal for 15 (2.68%) patients (4.24% of midlife adults and 15.62% of older adults). Older males were more likely to survive compared to females (100% vs. 66.67%, *p <* 0.0149).

**Figure 4:**
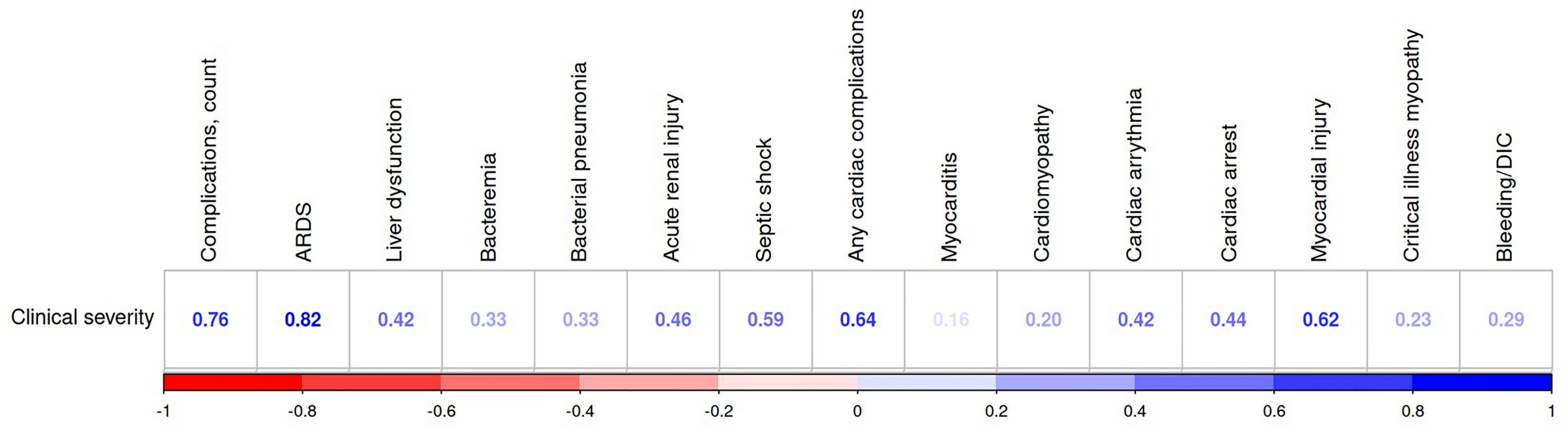
Association of clinical severity with COVID-19 complications (Dubai dataset). All the associations are significant (p<0.05). ARDC, acute respiratory distress syndrome; DIC, disseminated intravascular coagulation.

There was a noticeable positive correlation between the patients’ age and complications as well as between the latter and disease severity (see **Figure 4**). The most common complications that patients experienced were acute respiratory distress syndrome (ARDS)—76 (13.57%) cases, bacterial pneumonia—15 (2.68%), liver dysfunction—54 (9.64%), acute renal injury—47 (8.39%), septic shock—25 (4.46%), and seizure—5 (0.89%). Out of 123 (21.96%) patients who experience these complications, 17 were older adults (53.12% of the age group), 73—midlife adults (30.93%), and 33—young adults (11.3%). Young and midlife males were more likely to present complications than females from the same age groups (p-value ranges from 0.0012 to 0.0499). Older patients also experienced cardiac complications far more frequently: as cardiac arrhythmia (12.5% vs. 4.24% in midlife adults and 0.34% in and young adults; *p <* 0.001), cardiac arrest (12.5% vs. 4.24% in midlife adults; *p <* 0.001). There were significant associations (*p <* 0.001) between disease severity and the total number of complications (*r* = 0.76), ARDS (*r* = 0.82), any cardiac complication (*r* = 0.64), including myocarditis (*r* = 0.16), septic shock (*r* = 0.59), acute renal injury (*r* = 0.46), and liver dysfunction (*r* = 0.42).

There were marked differences in symptoms among age and sex groups. Cough and fever were more frequent symptoms in midlife adults (*p <* 0.001 and 0.0108), sputum and shortness of breath in older adults (*p* = 0.0228 and 0.001), and headache in young adults (*p* = 0.021). Symptoms such as sore throat, headache, and loss of smell or taste were more common in females (*p* = 0007, 0.0445, and 0.0396). The tendency was especially notable in young and midlife adults.

### 4.3 Forecast of COVID-19 Severity From Age and Sex Along With Laboratory Findings and Personal Risk Factors

The risk of the non-mild form of COVID-19 increased with advancing age considerably (see **Table 2**). From the radiological findings, the risk of the non-mild COVID-19 was 1.5 times higher in midlife adults (*aOR* = 1.52475; *p* = 0.01265) and 4.7 times greater in older adults (*aOR* = 4.72629; *p* = 0.02364) compared to young adults. This relationship was more pronounced when we analyzed the clinical markers of disease severity for midlife adults (*aOR* = 5.55; *p <* 0.001) and older adults (*aOR* = 17.05; *p <* 0.001) with young adults as a reference. Age disparities were more pronounced when studied with the clinical markers of disease severity than with the radiological markers. The higher sensitivity of the clinical markers for our study was even more evident in sex-related differences: men were more likely to develop a non-mild form of COVID-19 as per clinical classification (*aOR* = 1.82; *p* = 0.0083), but there was no strong argument to support this statement when the radiologic features were in use (*aOR* = 1.2; *p* = 0.44).

The multivariate analysis provided similar findings. In general, age and sex were important predictors of disease severity among the set of data typically collected on admission.

**Figure 5:**
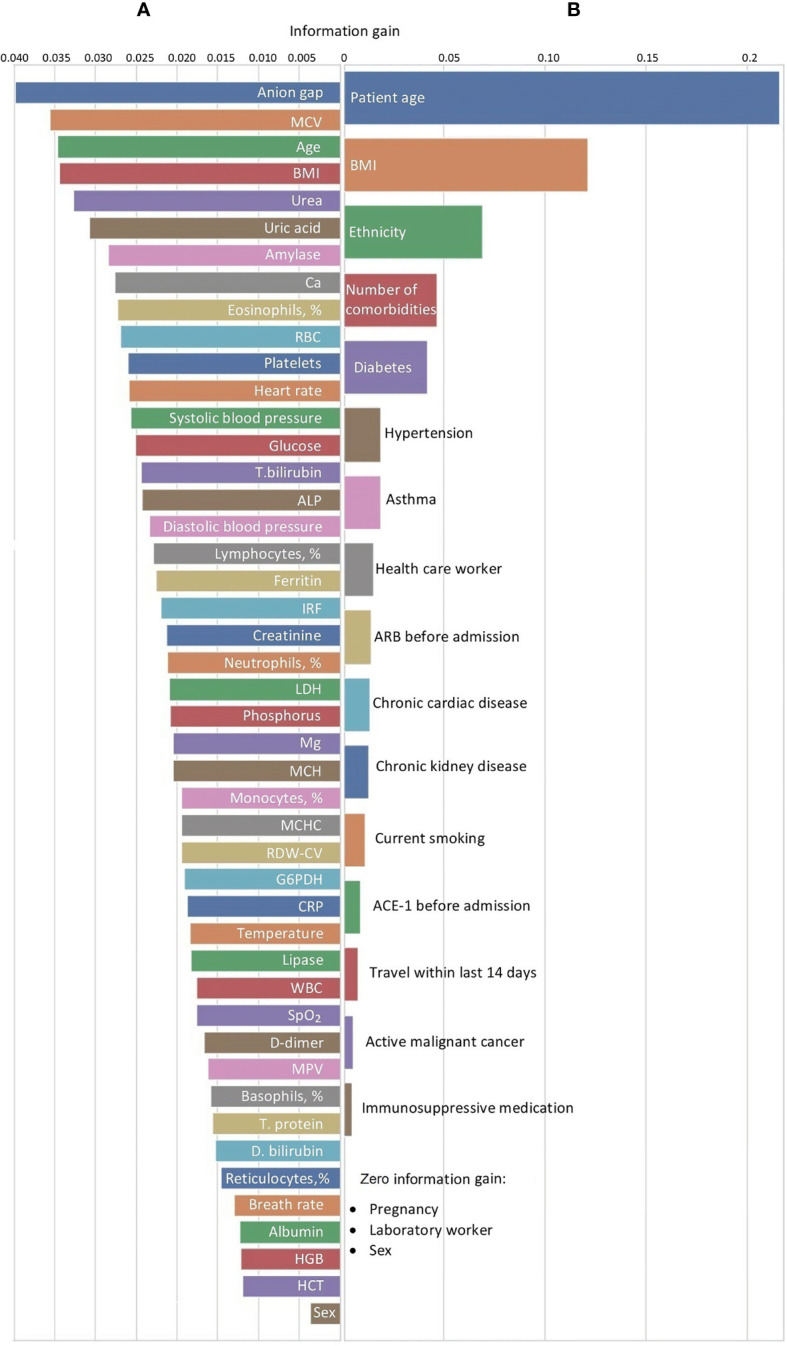
Feature selection for predicting disease severity assessed with **(A)** laboratory findings or **(B)** clinical criteria in Dubai dataset. ACE-I, angiotensin-converting-enzyme inhibitors; ALP, alkaline phosphatase; ARB, angiotensin-II receptor blockers; ARDS, acute respiratory distress syndrome; BMI, body mass index; CRP, C-reactive protein; D.bilirubin, direct bilirubin; G6PDH, glucose-6-phosphate dehydrogenase; GCS, Glasgow coma scale; GI, gastrointestinal; HGB, hemoglobin; HCT, hematocrit; ICU, intensive care unit; IRF, immature reticulocyte fraction; LDH, lactate dehydrogenase; MCV, mean corpuscular volume; MCH, mean corpuscular hemoglobin level; MCHC, mean corpuscular hemoglobin concentration; MPV, mean platelet volume; RBC, red blood cells; RDW-CV, red blood cell distribution width; SpO2, oxygen saturation; SOFA, sequential organ failure assessment; T. bilirubin, total bilirubin; T. protein, total protein; WBC, white blood cells.

The comparison of the informative value of age and sex with the laboratory findings showed age to be on the list of the top valuable predictors, and sex to be less important (see **Figure 5A**). Patients’ age was the most important feature among individual risk factors (see **Figure 5B**). In the list of predictors ranked by their informative gain, age was followed by BMI, ethnicity, total number of comorbidities and, finally, sex. Recently, we published a detailed analysis of ethnic disparities of COVID-19 (Al Zahmi et al., 2022).

**Figure 6:**
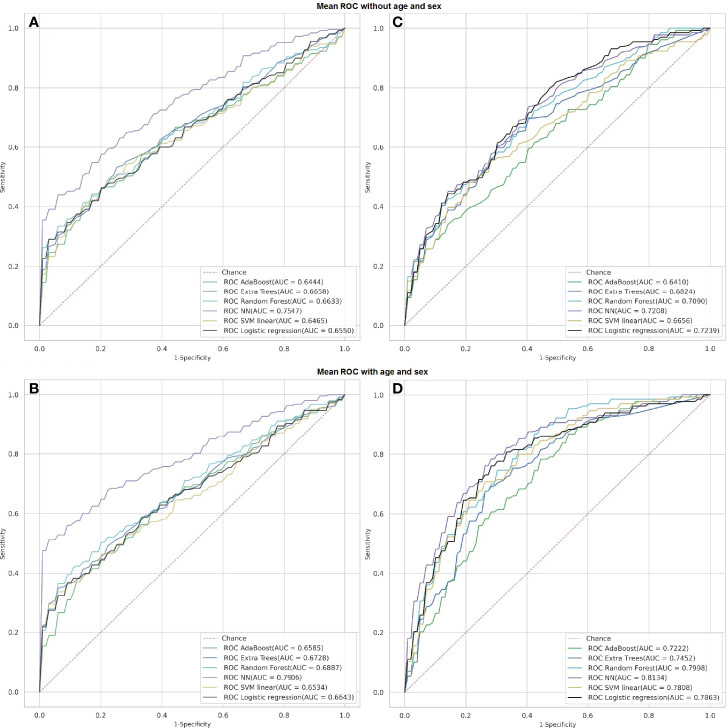
Receiver operating characteristics of classification models predicting severity of COVID-19 from laboratory findings (**A, B**; Abu Dhabi dataset) or individual risk factors (**C, D**; Dubai dataset) with and without age and sex.

The prediction of the disease progression was more accurate after considering sex and age of the individuals. The area under the curve value increased pronouncedly (*p <* 0.05) after we added these predictors to the model forecasting disease severity from individual risk factors (+8.41%; see **Table 3** and **Figure 6**).

## 5 Discussion

The results of our study indicate a higher severity of COVID-19 disease in men and in older patients. With regard to sex dissimilarities, the number of male patients exceeded the number of female patients in both study centers allocated in different Emirates. These findings are similar to the results of other studies on age and sex features of various infectious diseases held in the past. They show differences in the response to infectious diseases between sexes and among age groups. For example, a retrospective analysis of hospitalization for pneumonia and influenza illustrates higher rates of admission in men of all age groups. This is also true for patients aged 65 years and over (Crighton et al., 2007). Moreover, the coronavirus that caused MERS in 2012–2018 affected mostly men (Das et al., 2021) and in the UAE more than 70% patients diagnosed with MERS were males (Ahmadzadeh et al., 2020).

### 5.1 Risk Factors and Comorbidities Associated With Age and Sex

Our study reports hypertension as the most common comorbidity in COVID-19 patients followed by diabetes. Similar studies have reported an increased risk of mortality from COVID-19 in patients with underlying heart conditions, e.g., with hypertension in males over 55 years of age and with coronary artery disease in females under 65 years (Aghajani et al., 2021). Hypertension, obesity, and chronic kidney disease were the predictors of mortality in another study (Jun et al., 2021). Patients diagnosed with COVID-19 commonly suffer from such diseases as hypertension (62.9%), hyperlipidemia (47.7%), and diabetes (39.8%) (Guerson-Gil et al., 2021). Male patients tend to have more background diseases. The spectrum of the background diseases also differs between sexes. In a study, peripheral vascular disease, dementia, chronic pulmonary disease, connective tissue disease, peptic ulcer disease and malignancies were common in women suffering from COVID-19, whereas a history of hypertension, hemiplegia, or myocardial infarction was more typical of men infected with SARS-CoV-2 (Cho et al., 2021).

Obesity is a risk factor for admission to ICU and intubation for coronavirus pneumonia in both sexes (Jun et al., 2021). In the present study the mean BMI ranges were within the interval of 27–28 kg/m2 in both hospital cohorts. This refers to overweight or pre-obesity. On average, patients of both sexes aged above 65 years had a BMI greater than 31 kg/m2 indicating obesity. Recent studies suggested that the level of obesity correlated independently with the in-hospital mortality in patients with COVID-19. Moreover, people with a BMI above 25 kg/m2 are at an increased risk of developing severe pneumonia and the in-hospital mortality was higher in underweight patients and in individuals with morbid obesity (Guerson-Gil et al., 2021).

### 5.2 Radiologic Findings in COVID-19 With Regard to Age and Sex

In the patients with COVID-19, the involvement of the lung parenchyma varies between sexes and among ages. From our study, age bias in the lung involvement seemed to be stronger than sex bias (see **Figure 1**).

#### 5.2.1 Sex Differences in Radiologic Findings in COVID-19

The present study demonstrates a significant sex disparity of the lung involvement in young adults. The data reflect a more severe lung involvement in men, i.e., the lesions are denser in men compared to women of the same age (*p <* 0.05). Although we did not assess the distribution of lesions across the lung space, our findings are compliant with the results of recent studies on related issues (Dangis et al., 2020). In contrast to these studies, we resort to the analysis of radiomics features extracted from semiautomatic segmentation of the lung lesions and lobes because this approach seems to be the most comprehensive one.

A study on CT appearance of COVID-19 presents differences between male and female patients of two age groups: below and above 60 years. There is a considerable difference in the peripheral involvement of the lung lobes (100% in males vs. 88.2% in females) as well as in the peribronchovascular distribution of the lesions (18.2% vs. 41.2%). In the age group below 60 years, the anterior distribution of the lesions was more common in men (66.7% vs. 41.7% in women). In each lung lobe, the CT score was higher in male patients. This leads to higher numbers of the total lung CT score in men (11 vs. 6.5) (Moradi et al., 2020). Other scientists observed unilateral pneumonia with ground-glass opacification mostly in women. They detected bilateral pneumonia more often in men (Wang et al., 2021).

#### 5.2.2 Age-Related Dissimilarities in Radiologic Appearance of COVID-19

*Lung involvement* in COVID-19 in diverse age groups has only been studied in a few studies. Previous work reported the lung involvement at the level of 36.4% in the young group versus 67.9% in the group above 60 years of age (Zhu et al. 2020). These data are consistent with our findings which show the percentage of the lung involvement in young adults to be approximately twice as low as in midlife adults and three times as low as in older adults. In line with this, several studies show bilateral lung involvement to be more common in older adults (Li et al., 2020; Fan et al., 2020; Zhao et al., 2020). Single focal lesions are characteristic for patients under 35 years of age, diffuse ones for the individuals aged 60 years and over (Fan et al., 2020; Liu et al., 2020b).

Both sexes present with a marked association between age and such a metric as a “combined lung severity and pulmonary score.” Notably, men of the age range from 50 to 79 years portray a higher score value. Conversely, the score values were higher for females aged 80 years and over (Borghesi et al., 2020b). In another type of coronavirus pneumonia, scientists also observed a positive association between the chest radiographic score and the disease progression in patients diagnosed with MERS (Das et al., 2021). A recent study also provided evidence for a distinct age bias in asymptomatic and symptomatic patients with COVID-19. In symptomatic patients, the total lung score in the elderly was higher than in the young patients (8.5 vs. 5.0; p = 0.07). In contrast, the score did not differ significantly in the asymptomatic group (Mori et al., 2021). The value of the aforementioned studies is limited as almost all of them did not consider background diseases that may confound the results.

*The lung density*. In our study, the density of the lesions was higher in men and in older adults. Notably, men were more often in a severe or critical condition than women. According to a prior chest evaluation report on patients with COVID-19-associated pneumonia, critical cases had a higher mean density of the lung compared to the ordinary cases and this was most likely accounted for by interstitial changes (Lyu et al., 2020).

*The lung lobe involvement* is another radiomical feature under analysis. According to our data, the involvement of both right and left lower lobes at the early disease phase was approximately equal in both sexes and all ages. As the disease progresses, we noted age- and sex-specific differences in this feature. A supposed reason for this is that the lobar distribution of the ground glass opacities (GGO) reflects the COVID-19 progression. The right and left lower lobes were affected most commonly, particularly in 42% and 38% of cases, respectively. Other researchers have also shown that opacification in any lobe correlates with the risk of hospitalization and intubation except for the one allocated in the left lower lobe (Toussie et al., 2020). The lung opacities in COVID-19 are commonly bilateral, peripheral, and basilar in distribution. At the early stage of the disease, they predominantly occupy the right lower lobe predominantly (Shi et al., 2020). This finding can be related to the thick and short physiological structure of the right lower lobe bronchus, which allows the virus to enter this area easily (Chen et al., 2020). However, other research gives the opposite data that the left lower lobe is affected most commonly in all age groups (Li et al., 2020).

The total number of the affected lobes and the presence of mild pleural thickening correlates with the age of patients (Wang et al., 2020; Liu et al., 2020b). The lesions are distributed across multiple lobes, predominantly in the posterior and peripheral parts of the lungs (Wang et al., 2020).

Some authors show a profound age difference in the involvement of the peripheral parts of the right upper and middle lobes. They report such a difference between the young (<60 years) and older adult (*≥*60 years) symptomatic patients (Mori et al., 2021). This is in line with another study that shows a variance in distribution of lesions across subpleural parts of the lung. In the midlife patients, it is twice as large as in the young and older adults infected with SARS-CoV-2 (Fan et al., 2020).

*Lung lesions*. In our study, the portion of the lung volume occupied with GGO was higher than the one affected in the form of consolidation. This tendency was true for all the age groups and reflects the known dynamics of the radiologic changes over the disease course. Chest CT examinations in an in-patient clinic in Wuhan (China) elucidated distinct lung opacification patterns with regard to age. In the group below 60 years, the proportion of patients with consolidation, pure GGO, and GGO with consolidation in the peripheral area was 1: 2.1: 3.4. The ratio for the group above 60 years is 1: 2.5: 4.2, respectively (Zhu et al., 2020). There is a slight increase in consolidation of the inflammatory exudate in the alveoli of patients aged above 60 years (Fan et al., 2020). Researchers presume that the appearance of the pure GGO is slightly lower than that of its combination with consolidation. This happens because the pure GGO is present in the early stage of the disease. The old-age group is characterized by a higher proportion of extensive involvement of the lung lobes (71.4% vs. 36.4%, *p* = 0.009) and a higher incidence of subpleural line and pleural thickening (50.0% vs. 25.0%, and 71.4% vs. 40.9%, *p* = 0.030 and *p* = 0.011, respectively) (Zhu et al., 2020).

### 5.3 Biochemical Response to COVID-19 With Regard to Age and Sex

#### 5.3.1 Sex Disparities in Biochemical Response to COVID-19

*Biochemical substrates*. The results we observed on the study cohorts from hospitals in two Emirates support the findings obtained on a multinational database of 41 global healthcare organizations (Alkhouli et al., 2020). From the analysis, the level of stress and inflammatory markers (CRP, fasting glucose levels), the end products of protein metabolism (urea nitrogen, creatinine), and heme metabolism (bilirubin) are disproportionally higher in men. This presents sex disparities in the response to the disease. Both in our study and in the study based on the large multinational cohort, the level of albumin was slightly lower in men. The additive value of our study is the uniformly based sample consisting of all the patients admitted subsequently to the hospitals regardless of disease severity. This allows us to verify the aforementioned sex disparities and to justify their statistical significance.

*Biochemical enzymes*. In our study, the levels of ALT, AST, and LDH were higher in males. No significant difference between sexes was found for the level of alkaline phosphatase (AP). In addition, we did not observe considerable difference in the level of either LDH or AP. Despite this, the levels of amylase, lipase, and glucose-6-phosphate dehydrogenase (G6PDH) were markedly higher in men, thus justifying the disparities in metabolic changes between males and females. Similarly, a multinational study elucidated sex-related differences in the activity of serum enzymes (Alkhouli et al., 2020).

*Electrolytes*. Our data showed a pronounced difference in the blood level of potassium and bicarbonate, both reflecting a more severe level of hypoxia in men. This may be well explained by the sex dissimilarity in the lung involvement seen on CT (see **Table 4**). The multinational registry shows the concentration of bicarbonate to be approximately equal in both sexes and the level of sodium to be slightly higher in men. Also, there was no considerable difference in calcium, magnesium, and chloride levels in that study (Alkhouli et al., 2020). Thus, the electrolyte disbalance provides evidence for the respiratory changes that are more pronounced in male patients.

*Hematologic findings*. No notable differences related to COVID-19 were found between the sexes in hematological findings either in our study or in the broad study that involved 6,387 men and 8,325 women (Alkhouli et al., 2020). The same was true for the platelets and the WBC count (Alkhouli et al., 2020). However, another study on sex differences in the immune resistance to SARS-CoV-2 found an association of the weak T cell response with worse disease outcome in males. Conversely, high innate levels of cytokines (e.g., IL-1, IL-6) were predictive of a mild disease form in women (Takahashi et al., 2020). Cytokines rise in patients diagnosed with COVID-19, and the levels of IL-4 and sCD40L cytokines were higher in men than in women in our study. The association between these types of cytokines shows differences between female and male cytokine responses (Petrey et al., 2021). A recent study conducted in China showed higher lymphocyte and lipoprotein levels in females (Sha et al., 2021).

*Clinical symptoms*. A few facts are found on the variance in the clinical appearance of COVID-19 with regard to age (Liu et al., 2020b). In our study, a sore throat and headache in women are almost twice as common as in men (40.21 vs. 25.88%; *p <* 0.0007 and 24.87 vs. 17.52%; *p <* 0.0445). A similar study reported fatigue at the level of 11% in the elderly and only 7% in the younger and middle-age groups (Liu et al., 2020a).

#### 5.3.2 Dissimilarities in the Response to COVID-19 Among Age Groups

*Coagulogram*. Much research has been done to find out what accounts for a larger number of complications and a higher mortality rate in the elderly. Some manage to link age with procoagulant changes in the blood clotting system. For instance, a few studies reported that coagulation deviations can be associated with older age (Sayad and Rahimi, 2020). Such coagulogram abnormalities may lead to thrombotic complications and endanger infected individuals even more (Giannis et al., 2020). A comparison of two age groups (below and above 60 years old) shows a markedly higher APTT level in the older group. Patients aged above 60 years had a higher level of fibrinogen than in the younger group (Peng et al., 2021). A conflicting finding in a study from Tehran does not seem to be representative because of a small sample size. In that study of patients with severe COVID-19, the fibrinogen level was 4.9 *±* 1.9 g/l in 6 patients aged less than 60 years versus 2.4 *±* 0.6 g/l in 10 patients aged above 60 years (*p* = 0.08) (Sayad and Rahimi, 2020). Researchers observed a positive association of the level of D-dimer with advancing age (Zhao et al., 2020; Peng et al., 2021). However, the above mentioned Iranian study reported an opposite tendency (Sayad and Rahimi, 2020).

*Biochemical findings*. The general outcome of our research and other studies is that the age-related disparities in biochemical findings are analogous to the sex-related dissimilarities. The risk of metabolic dysregulation is higher in older adults than in young adults. In an equivalent way, male sex is associated with abnormal levels of a set of biochemical parameters. For instance, a study showed a significantly lower level of *troponin* in the younger group (Peng et al., 2021). There was an age-related bias in the level of *electrolytes* indicative of hypoxia (e.g., potassium) (Zhao et al., 2020; Peng et al., 2021).

*Laboratory markers of the protein metabolism* have evident age-related bias in COVID-19. The levels of total protein and albumin are significantly higher in younger patients (Zhao et al., 2020; Liu et al., 2021; Peng et al., 2021). Contrarily, in the older patients researchers noted a significantly higher concentration of the end products of the protein and heme metabolism, i.e., urea (Peng et al., 2021), blood urea nitrogen (Zhao et al., 2020; Liu et al., 2021), creatinine (Zhao et al., 2020; Peng et al., 2021), total bilirubin (Zhao et al., 2020; Peng et al., 2021), and direct bilirubin (Zhao et al., 2020). However, there are some conflicting findings on the association of the total bilirubin (Liu et al., 2021) and direct bilirubin (Peng et al., 2021) with advancing age. Besides the difference in disease severity, an appreciably higher glomerular filtration rate in the young patients may explain these facts (Liu et al., 2021).

*Enzymatic activity* is subject to age. However, there are conflicting findings on the activity of ALT (Liu et al., 2020b; Liu et al., 2021; Peng et al., 2021; AST Zhao et al., 2020; Liu et al., 2021; Peng et al., 2021), ALP (Liu et al., 2021), LDH (Peng et al., 2021), GGTP (Liu et al., 2021), and CK (Zhao et al., 2020; Liu et al., 2021; Peng et al., 2021). Supposedly, the high variance in the individual enzymatic activity induced by pathology accounts for the discrepancies among independent studies.

*Inflammatory markers*. In analogy to our study, other studies justify age and sex as valuable predictors of disease severity with distinct approaches to data analysis. Similar to our study, the level of CRP on admission and during the disease course was higher in the older patients (Zhao et al., 2020; Peng et al., 2021). CRP fluctuations are independent risk factors for ICU admission in young and middle-aged severe patients in contrast to the older ones (Liu et al., 2021).

*Cytokines*. In our study, the individual variance in the level of interleukin-6 (IL-6) was considerable among patients. A recent study found evidence to suggest that elderly patients were prone to a cytokine storm (Xu et al., 2021). Because of the high variance, there are conflicting findings on whether the age difference in IL-6 is notable (Zhao et al., 2020) or not (Peng et al., 2021). A study shows greater pulmonary concentrations of proinflammatory cytokines in men than in women (Conti and Younes, 2020). The opposite tendency was observed in influenza A infection (Morgan and Klein, 2019). Further studies on this issue are required to verify the results.

**Hematologic findings**. *Platelets*. From our data, the platelet count did not vary among the age groups. In a study conducted in China, an absolute count of platelets did not differ considerably in patients of different ages (Peng et al., 2021). The same tendency was observed in severe patients of two age groups with a threshold of 60 years (Liu et al., 2021). Identical results were reported by a research group from Iran (Sayad and Rahimi, 2020). In a study that included patients of three age groups (<60 years; 60–74 years; *≥*75 years), the middle-aged patients had a remarkably higher platelet count compared to the younger ones (215 vs. 209), whereas the oldest patients showed the lowest (186) platelet count (Zhao et al., 2020).

*WBC*. We did not observe any associations between WBC and age; however, one study reported a marked increase in the WBC count with advancing age (Zhao et al., 2020), while others failed to observe this age-related pattern in WBC (Peng et al., 2021); Liu et al., 2021). Despite the discrepancies, the mean leukocyte count for the groups ranges within the reference diapason. Thus, the dissimilarities have no clinical value.

**Neutrophils and monocytes** are an issue in our study as the data on them are also discrepant. Previous research has reported that either the absolute count of neutrophils and monocytes was not associated with age (Peng et al., 2021) or it rises with advancing age (Zhao et al., 2020; Liu et al., 2021). Our findings support the second statement.

*The absolute lymphocyte count* has been shown to be negatively associated with age (Zhao et al., 2020; Liu et al., 2021; Peng et al., 2021). Consistently, a retrospective study reported a markedly lower percentage of lymphocytes in the older group 19.15 (10.58–26.93) than in the young and middle-aged group 28.95 (24.45–33.58) (Liu et al., 2020a). Another reference shows an appreciably lower percentage of lymphocytes in older adults compared to younger adult patients (24.4% vs. 26.5%; *p* = 0.02) (Mori et al., 2021).

*The red blood cells count and the level of hemoglobin* decreased with advancing age in our study as well as in reports of other studies (Liu et al., 2020b). However, another study reported a slight difference in the indices across the age (Peng et al., 2021).

### 5.4 Informative Value of Sex and Age in Prediction of COVID-19 Severity

In analogy to our findings, other studies with distinct approaches to data analysis have reported evidence to suggest age and sex as valuable predictors of disease severity. In a study on prediction of the in-hospital mortality for COVID-19, researchers highlighted that the patients’ age conferred the highest risk of death. Furthermore, a multivariate analysis shows that age along with a CXR severity index and immunosuppression were strongly associated with the in-hospital mortality (Borghesi et al., 2020a). Similarly, older adults were more frequently affected during a MERS outbreak in Saudi Arabia, and the 30-days mortality was higher for that age group Ahmed (2017).

A low oxygen saturation level (*SpO*_2_) is a known predictor of mortality or a rapid progression of COVID-19 to more severe forms requiring hospitalization and ICU admission (Covino et al., 2020; Xie et al., 2020). To forecast an adverse outcome in COVID-19, previous researchers built a multivariate model based on age and other covariates (e.g., oxygen saturation, asthma). The accuracy of the model was lower than in our study, and the authors did not assess the informative value of age in the prediction (Goodacre et al., 2021). Scientists and clinical specialists observe high severity of the disease or death mostly in patients with the oxygenation level below 90%. For example, a retrospective analysis of clinical data of patients from China indicates that the *SpO*_2_ level below or equal to 90% is a predictor of the severe and critical disease. According to the study, older patients (61–78 years) had a lower oxygenation status compared to the middle-aged adults (40–63 years) (Xie et al., 2020). In another study, the saturation level correlated negatively with age. Patients in a severe or critical condition have a mean *SpO*_2_ level of 90.25%, while in other cases it is 97% (Mi et al., 2020). Another research group states that the oxygen saturation on admission below 90% is strongly associated with the in-hospital mortality for COVID-19, but the *SpO*_2_ level was not age dependent (Mejía et al., 2020).

Inconsistent findings and models with weak performance justify a necessity for a more precise analysis that would elucidate the impact of age on the level of oxygen saturation which is predictive of disease outcomes (Goodacre et al., 2021).

## 6 Strength and Limitations

There were several strengths to the study. Firstly, the study cohort was representative of the COVID-19 population in Dubai including all adult age groups and disease severity enabling us to calculate actual risk estimates. *Secondly*, it was a multicentered study which compared data from two hospitals functioning in separate Emirates. Hospitals in each Emirate report to a designated health authority of a distinct emirate, i.e., Dubai Health Authority and Department of Health of Abu Dhabi. This justifies them as independent centers for clinical services and studies. At the same time, the diagnostics and treatment were performed in full accordance with the common “National Guidelines for Clinical Management and Treatment of COVID-19” (National Emergency Crisis and Disasters Management Authority, 2020).

*The main limitation* of our study was the retrospective design. For this reason, we could not unify the settings of the study for both hospitals and collected the data available in their medical information systems.

*Another limitation* is that we resorted to different ways of assessing disease severity in the datasets used. Thus, the impact of age and sex on disease severity can be assessed only within the framework of each sample. However, the level of severity is not transferable between the datasets used.

## 7 Conclusion

Our study highlights several important novel findings that have clear clinical implications for the treatment and management of COVID-19 patients across a spectrum of age groups and disease severity:

- The need for 0_2_ supplementation was directly correlated with age. Intensive care was required more often for men than for women of all ages (*p <* 0.01). These facts mirror the results of biochemical findings and may justify a direct correlation of older age and male sex with a severe course of the disease.
- Laboratory data justify the trend toward worsening of the SARS-CoV-2 infection with age. The portion of mild cases decreases dramatically with age while the percentage of severe cases rises. Biochemical findings in both datasets support the tendencies identified from radiomics. Sexual dissimilarities were pronounced in young adults and reduced with age.
- The severity of COVID-19 assessed from the percentage of the total lung involvement did not differ between sexes. However, in young male adults, both the percentage of the lung parenchyma covered with consolidation and the density characteristics of lesions were higher. These facts suggest a more severe lung involvement in men. In midlife and older adults, no marked differences between sexes were found.
- From the univariate analysis, the risk of the non-mild COVID-19 was higher in midlife adults and older adults compared to young adults. Age disparities were more pronounced if studied with the clinical markers of disease severity than with the radiological markers. The higher sensitivity of clinical markers in our study is even more evident in sex-related differences. The multivariate analysis provides similar findings.
- Age and sex should be considered while forecasting the severity of COVID-19. This can improve management of patients, personnel, and equipment in real clinical settings.

## Data Availability Statement

The datasets presented in this study can be found in online repositories. The datasets generated for this study are available on request at the site of Big Data Analytics Center https://bi-dac.com.

## Ethics Statement

The studies involving human participants were reviewed and approved by the Department of Health Abu Dhabi (reference number: DOH/CVDC/2020/889), Mediclinic Middle East Research and Ethics Committee (reference number MCME.CR.104.MPAR.2020), and Dubai Scientific Research Ethics Committee of Dubai Health Authority (protocol number DSREC-05/2020_25). The committees approved the study for the retrospective analysis of the data obtained as a standard of care. No potentially identifiable personal information is presented in the study. Written informed consent for participation was not required for this study in accordance with the national legislation and the institutional requirements.

## Author Contributions

All authors contributed to the creation of the article as follows: JAK, FAZ, and YS formulated the objectives. JAK, FAZ, RA, HE, MaL, NS, RS, DK, and SN collected the datasets. YS, DS, and GS wrote the manuscript; TH performed the statistical analysis, prepared the figures and tables for data presentation and illustration, KG, MiL, TL, TA, AB and AD contributed to the literature review and data analysis. All the authors have reviewed the manuscript and vouched for the accuracy and completeness of the data. All authors contributed to the article and approved the submitted version.

## Funding

This work was supported by MBRU Collaborative Research Award 20-0081 (PI: KM Das), UAEU CMHS research grant (PI: KG).

## Conflict of Interest

The authors declare that the research was conducted in the absence of any commercial or financial relationships that could be construed as a potential conflict of interest.

## Publisher’s Note

All claims expressed in this article are solely those of the authors and do not necessarily represent those of their affiliated organizations, or those of the publisher, the editors and the reviewers. Any product that may be evaluated in this article, or claim that may be made by its manufacturer, is not guaranteed or endorsed by the publisher.

## Abbreviations

ACE-I, angiotensin-converting-enzyme inhibitors; adm, admission to the hospital; ALP, alkaline phosphatase; ALT, alanine aminotransferase; APTT, activated partial thromboplastin time; ARB, angiotensin-II receptor blockers; ARDS, acute respiratory distress syndrome; AST, aspartate aminotransferase; AUC, area under the curve; BMI, body mass index; CK, creatine kinase; CRRT, continuous renal replacement therapy; DIC, disseminated intravascular coagulation GCS, Glasgow coma scale; G6PDH, glucose-6-phosphate dehydrogenase; GGO, ground glass opacity; GGTP, gamma-glutamyl transpeptidase; GI, gastrointestina; HD, hemodialysis; HIV, human immunodeficiency virus; ICU, intensive care unit; IL-6, interleukin-6; LDH, lactate dehydrogenase; LMWH, low molecular weighted heparins; MERS-CoV, Middle East respiratory syndrome coronavirus; NN, neural network; NSAID, non-steroid anti-inflammatory drugs; PCR, reverse transcription-polymerase chain reaction; RBC, red blood cells; ROC, receiver operating characteristic; SD, standard deviation; SOFA, sequential organ failure assessment; SOB, shortness of breath; SpO2, oxygen saturation; WBC, white blood cells.

## References

[B1] Aghajani M. H. Asadpoordezaki Z. Haghighi M. Pourhoseingoli A. Taherpour N. Toloui A.. (2021). Effect of Underlying Cardiovascular Disease on the Prognosis of Covid-19 Patients; a Sex and Age-Dependent Analysis. Arch. Acad. Emerg. Med. 9, e65. doi: 10.22037/aaem.v9i1.1363 34870231 PMC8628643

[B2] Ahmadzadeh J. Mobaraki K. Mousavi S. J. Aghazadeh-Attari J. Mirza-Aghazadeh-Attari M. Mohebbi I. (2020). The Risk Factors Associated With Mers-Cov Patient Fatality: A Global Survey. Diagn. Microbiol. Infect. Dis. 96, 114876. doi: 10.1016/j.diagmicrobio.2019.114876 31959375 PMC7126953

[B3] Ahmed A. E. (2017). The Predictors of 3-and 30-Day Mortality in 660 Mers-Cov Patients. BMC Infect. Dis. 17, 615. doi: 10.1186/s12879-017-2712-2 28893197 PMC5594447

[B4] Alkhouli M. Nanjundappa A. Annie F. Bates M. C. Bhatt D. L. (2020). Sex Differences in Case Fatality Rate of Covid-19: Insights From a Multinational Registry. In Mayo Clin. Proc. (Elsevier) 95, 1613–1620. doi: 10.1016/j.mayocp.2020.05.014 PMC7256502 32753136

[B5] Alon T. M. Doepke M. Olmstead-Rumsey J. Tertilt M. (2020). The Impact of COVID-19 on Gender Equality. Tech. Rep. Natl. Bureau Econ. Res. doi: 10.3386/w26947

[B6] Al Zahmi F. Statsenko Y. Habuza T. Awawdeh R. Elshekhali H. Lee M.. (2022). Ethnicity- Specific Features of Covid-19 Among Arabs, Africans, South Asians, East Asians and Caucasians in the UAE. Front. Cell. Infect. Microbiol. 1241. doi: 10.3389/fcimb.2021.773141 PMC8967254 35368452

[B7] Bertsimas D. Lukin G. Mingardi L. Nohadani O. Orfanoudaki A. Stellato B.. (2020). Covid-19 Mortality Risk Assessment: An International Multi-Center Study. PLoS One 15 (12), e0243262. doi: 10.1371/journal.pone.0243262 33296405 PMC7725386

[B8] Borghesi A. Zigliani A. Golemi S. Carapella N. Maculotti P. Farina D.. (2020a). Chest X-Ray Severity Index as a Predictor of in-Hospital Mortality in Coronavirus Disease 2019: A Study of 302 Patients From Italy. Int. J. Infect. Dis. 96, 291–293. doi: 10.1016/j.ijid.2020.05.021 32437939 PMC7207134

[B9] Borghesi A. Zigliani A. Masciullo R. Golemi S. Maculotti P. Farina D.. (2020b). Radiographic Severity Index in Covid-19 Pneumonia: Relationship to Age and Sex in 783 Italian Patients. La Radiol. Med. 125, 461–464. doi: 10.1007/s11547-020-01202-1 PMC7194500 32358691

[B10] Chen Z. Fan H. Cai J. Li Y. Wu B. Hou Y.. (2020). High-Resolution Computed Tomography Manifestations of Covid-19 Infections in Patients of Different Ages. Eur. J. Radiol. 126, 108972. doi: 10.1016/j.ejrad.2020.108972 32240913 PMC7102649

[B11] Cho K. H. Kim S. W. Park J. W. Do J. Y. Kang S. H. (2021). Effect of Sex on Clinical Outcomes in Patients With Coronavirus Disease: A Population-Based Study. J. Clin. Med. 10, 38. doi: 10.3390/jcm10010038 PMC7794723 33374452

[B12] Colombi D. Bodini F. C. Petrini M. Maffi G. Morelli N. Milanese G.. (2020). Well-Aerated Lung on Admitting Chest Ct to Predict Adverse Outcome in Covid-19 Pneumonia. Radiology 296, E86–E96. doi: 10.1148/radiol.2020201433 32301647 PMC7233411

[B13] Conti P. Younes A. (2020). Coronovirus COV-19/SARSCoV-2 Affects Women Less Than Men: Clinical Response to Viral Infection. J. Biol. Regul. Homeost. Agents 34 (2), 339–343. doi: 10.23812/Editorial-Conti-3 32253888

[B14] Costagliola G. Spada E. Consolini R. (2021). Age-Related Differences in the Immune Response Could Contribute to Determine the Spectrum of Severity of Covid-19. Immun. Inflamm. Dis. 9, 331–339. doi: 10.1002/iid3.404 33566457 PMC8014746

[B15] COVID-19 CT Segmentation Dataset. (2021). Available at: https://www.medseg.ai [Accessed July 10, 2021].

[B16] Covino M. De Matteis G. Santoro M. Sabia L. Simeoni B. Candelli M.. (2020). Clinical Characteristics and Prognostic Factors in Covid-19 Patients Aged *Geq* 80 Years. Geriatr. Gerontol. Int. 20, 704–708. doi: 10.1111/ggi.13960 32516861 PMC7300699

[B17] Crighton E. Elliott S. Moineddin R. Kanaroglou P. Upshur R. (2007). An Exploratory Spatial Analysis of Pneumonia and Influenza Hospitalizations in Ontario by Age and Gender. Epidemiol. Infect. 135, 253–261. doi: 10.1017/S095026880600690X 16824252 PMC2870578

[B18] Dangis A. De Brucker N. Heremans A. Gillis M. Frans J. Demeyere A.. (2020). Impact of Gender on Extent of Lung Injury in Covid-19. Clin. Radiol. 75, 554. doi: 10.1016/j.crad.2020.04.005 32359867 PMC7177134

[B19] Das K. M. Alkoteesh J. A. Sheek-Hussein M. Alzadjali S. A. Alafeefi M. T. Singh R.. (2021). Role of Chest Radiograph in Mers-Cov Pneumonia: A Single Tertiary Referral Center Experience in the United Arab Emirates. Egypt. J. Radiol. Nucl. Med. 52, 1–7. doi: 10.1186/s43055-021-00517-x

[B20] Deeb A. Khawaja K. Sakrani N. AlAkhras A. Al Mesabi A. Trehan R.. (2021). Impact of Ethnicity and Underlying Comorbidity on Covid-19 Inhospital Mortality: An Observational Study in Abu Dhabi, Uae. BioMed. Res. Int. 2021, 6695707. doi: 10.1155/2021/6695707 33708993 PMC7930915

[B21] Dudley J. P. Lee N. T. (2020). Disparities in Age-Specific Morbidity and Mortality From Sars-Cov-2 in China and the Republic of Korea. Clin. Infect. Dis. 71, 863–865. doi: 10.1093/cid/ciaa354 32232322 PMC7184419

[B22] Fan N. Fan W. Li Z. Shi M. Liang Y. (2020). Imaging Characteristics of Initial Chest Computed Tomography and Clinical Manifestations of Patients With Covid-19 Pneumonia. Japanese J. Radiol. 38, 533–538. doi: 10.1007/s11604-020-00973-x PMC7171599 32318916

[B23] Giannis D. Ziogas I. A. Gianni P. (2020). Coagulation Disorders in Coronavirus Infected Patients: Covid-19, Sars-Cov-1, Mers-Cov and Lessons From the Past. J. Clin. Virol. 127, 104362. doi: 10.1016/j.jcv.2020.104362 32305883 PMC7195278

[B24] Goodacre S. Thomas B. Lee E. Sutton L. Loban A. Waterhouse S.. (2021). Post-Exertion Oxygen Saturation as a Prognostic Factor for Adverse Outcome in Patients Attending the Emergency Department With Suspected Covid-19: A Substudy of the Priest Observational Cohort Study. Emerg. Med. J. 38, 88–93. doi: 10.1136/emermed-2020-210528 33273040 PMC7716294

[B25] Guan W.-j. Liang W.-h. Zhao Y. Liang H.-r. Chen Z.-s. Li Y.-m.. (2020). Comorbidity and Its Impact on 1590 Patients With Covid-19 in China: A Nationwide Analysis. Eur. Respir. J. 55, 2000547. doi: 10.1183/13993003.00547-2020 32217650 PMC7098485

[B26] Guerson-Gil A. Palaiodimos L. Assa A. Karamanis D. Kokkinidis D. Chamorro-Pareja N.. (2021). Sex-Specific Impact of Severe Obesity in the Outcomes of Hospitalized Patients With Covid-19: A Large Retrospective Study From the Bronx, New York. Eur. J. Clin. Microbiol. Infect. Dis. 40, 1963–1974. doi: 10.1007/s10096-021-04260-z 33956286 PMC8101338

[B27] Habuza T. Khalil K. Zaki N. Alnajjar F. Gochoo M. (2020). “Web-Based Multi-User Concurrent Job Scheduling System on the Shared Computing Resource Objects,” in 2020 14th International Conference on Innovations in Information Technology (IIT) (IEEE), Al Ain, UAE, 221–226. doi: 10.1109/IIT50501.2020.9299110

[B28] Habuza T. Navaz A. N. Hashim F. Alnajjar F. Zaki N. Serhani M. A.. (2021). AI Applications in Robotics, Precision Medicine, and Medical Image Analysis: An Overview and Future Trends. Inf. Med. Unlocked 24, 100596. doi: 10.1016/j.imu.2021.100596

[B29] Hariyanto T. I. Valeriani K. Kwenandar F. Damay V. Siregar J. Lugito N. P. H.. (2020). Inflammatory and Hematologic Markers as Predictors of Severe Outcomes in Covid-19 Infection: A Systematic Review and Meta-Analysis. Am. J. Emerg. Med. 41, 110–119. doi: 10.1016/j.ajem.2020.12.076 33418211 PMC7831442

[B30] Harrison E. M. Docherty A. B. Barr B. Buchan I. Carson G. Drake T. M.. (2020). Ethnicity and Outcomes From Covid-19: The Isaric Ccp-Uk Prospective Observational Cohort Study of Hospitalised Patients. doi: 10.2139/ssrn.3618215

[B31] Hofmanninger J. Prayer F. Pan J. Röhrich S. Prosch H. Langs G. (2020). Automatic Lung Segmentation in Routine Imaging Is Primarily a Data Diversity Problem, Not a Methodology Problem. Eur. Radiol. Exp. 4, 1–13. doi: 10.1186/s41747-020-00173-2 32814998 PMC7438418

[B32] Hsu H. E. Ashe E. M. Silverstein M. Hofman M. Lange S. J. Razzaghi H.. (2020). Race/Ethnicity, Underlying Medical Conditions, Homelessness, and Hospitalization Statusof Adult Patients With COVID-19 at an Urban Safety-Net Medical Center - Boston, Massachusetts, 2020. MMWR Morb. Mortal. Wkly. Rep. 69, 864. doi: 10.15585/mmwr.mm6927a3 32644981 PMC7727597

[B33] Islam N. Khunti K. Dambha-Miller H. Kawachi I. Marmot M. (2020). Covid-19 Mortality: A Complex Interplay of Sex, Gender and Ethnicity. Eur. J. Public Health 30, 847–848. doi: 10.1093/eurpub/ckaa150 32745211 PMC7545966

[B34] Jenkinson M. Beckmann C. F. Behrens T. E. Woolrich M. W. Smith S. M. (2012). Fsl. Neuroimage 62, 782–790. doi: 10.1016/j.neuroimage.2011.09.015 21979382

[B35] Jin J.-M. Bai P. He W. Wu F. Liu X.-F. Han D.-M.. (2020). Gender Differences in Patients With Covid-19: Focus on Severity and Mortality. Front. Public Health 8, 152. doi: 10.3389/fpubh.2020.00152 32411652 PMC7201103

[B36] Jones T. C. Biele G. Mühlemann B. Veith T. Schneider J. Beheim-Schwarzbach J.. (2021). Estimating Infectiousness Throughout Sars-Cov-2 Infection Course. Science 373, eabi5273. doi: 10.1126/science.abi5273 34035154 PMC9267347

[B37] Jun T. Nirenberg S. Weinberger T. Sharma N. Pujadas E. Cordon-Cardo C.. (2021). Analysis of Sex-Specific Risk Factors and Clinical Outcomes in Covid-19. Commun. Med. 1, 1–8. doi: 10.1038/s43856-021-00006-2 PMC9053255 35602223

[B38] Klein S. L. (2012). Sex Influences Immune Responses to Viruses, and Efficacy of Prophylaxis and Treatments for Viral Diseases. Bioessays 34, 1050–1059. doi: 10.1002/bies.201200099 23012250 PMC4120666

[B39] Lee P.-I. Hu Y.-L. Chen P.-Y. Huang Y.-C. Hsueh P.-R. (2020). Are Children Less Susceptible to Covid-19? J. Microbiol. Immunol. Infect. 53, 371–372. doi: 10.1016/j.jmii.2020.02.011 32147409 PMC7102573

[B40] Li W. Fang Y. Liao J. Yu W. Yao L. Cui H.. (2020). Clinical and Ct Features of the Covid-19 Infection: Comparison Among Four Different Age Groups. Eur. Geriatr. Med. 11, 843–850. doi: 10.1007/s41999-020-00356-5 32662041 PMC7355129

[B41] Liu K. Chen Y. Lin R. Han K. (2020a). Clinical Features of Covid-19 in Elderly Patients: Acomparison With Young and Middle-Aged Patients. J. Infect. 80, e14–e18. doi: 10.1016/j.jinf.2020.03.005 PMC7102640 32171866

[B42] Liu X. Lv J. Gan L. Zhang Y. Sun F. Meng B.. (2020b). Comparative Analysis of Clinical Characteristics, Imaging and Laboratory Findings of Different Age Groups With Covid-19. Indian J. Med. Microbiol. 38, 87–93. doi: 10.4103/ijmm.IJMM_20_133 32719214 PMC7706422

[B43] Liu Z. Wu D. Han X. Jiang W. Qiu L. Tang R.. (2021). Different Characteristics of Critical Covid-19 and Thinking of Treatment Strategies in non-Elderly and Elderly Severe Adult Patients. Int. Immunopharmacol. 92, 107343. doi: 10.1016/j.intimp.2020.107343 33450596 PMC7833421

[B44] Luo H. Liu S. Wang Y. Phillips-Howard P. A. Ju S. Yang Y.. (2020). Age Differences in Clinical Features and Outcomes in Patients With Covid-19, Jiangsu, China: A Retrospective, Multicentre Cohort Study. BMJ Open 10, e039887. doi: 10.1136/bmjopen-2020-039887 PMC7536631 33020106

[B45] Lu X. Zhang L. Du H. Zhang J. Li Y. Y. Qu J.. (2020). Sars-Cov-2 Infection in Children. N. Engl. J. Med. 382, 1663–1665. doi: 10.1056/NEJMc2005073 32187458 PMC7121177

[B46] Lyu P. Liu X. Zhang R. Shi L. Gao J. (2020). The Performance of Chest Ct in Evaluating the Clinical Severity of Covid-19 Pneumonia: Identifying Critical Cases Based on Ct Characteristics. Invest. Radiol. 55, 412–421. doi: 10.1097/RLI.0000000000000689 32304402 PMC7173027

[B47] Mejía F. Medina C. Cornejo E. Morello E. Vásquez S. Alave J.. (2020). Oxygen Saturation as a Predictor of Mortality in Hospitalized Adult Patients With Covid-19 in a Public Hospital in Lima, Peru. PLoS One 15, e0244171. doi: 10.1371/journal.pone.0244171 33370364 PMC7769479

[B48] Mi J. Zhong W. Huang C. Zhang W. Tan L. Ding L. (2020). Gender, Age and Comorbi- Dities as the Main Prognostic Factors in Patients With Covid-19 Pneumonia. Am. J. Trans. Res. 12, 6537. PMC7653634 33194050

[B49] Moradi B. Ghanaati H. Kazemi M. A. Gity M. Hashemi H. Davari-Tanha F.. (2020). Implications of Sex Difference in Ct Scan Findings and Outcome of Patients With Covid-19 Pneumonia. Radiol.: Cardiothorac. Imaging 2, e200248. doi: 10.1148/ryct.2020200248 33778607 PMC7370352

[B50] Morgan R. Klein S. L. (2019). The Intersection of Sex and Gender in the Treatment of Influenza. Curr. Opin. Virol. 35, 35–41. doi: 10.1016/j.coviro.2019.02.009 30901632 PMC6556398

[B51] Mori H. Obinata H. Murakami W. Tatsuya K. Sasaki H. Miyake Y.. (2021). Comparison of Covid-19 Disease Between Young and Elderly Patients: Hidden Viral Shedding of Covid-19. J. Infect. Chemother. 27, 70–75. doi: 10.1016/j.jiac.2020.09.003 32950393 PMC7474868

[B52] National Emergency Crisis and Disasters Management Authority. (2020). Natio- Nal Guidelines for Clinical Management and Treatment of Covid-19- Version 4.1. Available at: https://www.dha.gov.ae/en/HealthRegulation/Documents/National_Guidelines_of_COVID_19_1st_June_2020.pdf (Accessed 01/08/2020).

[B53] Pagano M. T. Peruzzu D. Ruggieri A. Ortona E. Gagliardi M. C. (2020). Vitamin D and Sex Differences in Covid-19. Front. Endocrinol. 11, 788. doi: 10.3389/fendo.2020.567824 PMC7554594 33101200

[B54] Peckham H. de Gruijter N. M. Raine C. Radziszewska A. Ciurtin C. Wedderburn L. R.. (2020). Male Sex Identified by Global Covid-19 Meta-Analysis as a Risk Factor for Death and Itu Admission. Nat. Commun. 11, 1–10. doi: 10.1038/s41467-020-19741-6 33298944 PMC7726563

[B55] Peng Q.-Y. Ma X.-H. Liu Z.-Y. Zhao C.-G. Zhang L. Qian Z.-X.. (2021). Differences in Clinical Characteristics Between Younger and Older Patients With Covid-19 and Their Relationship With the Length of Hospital Stay. J. Intensive Med. 1, 123–129. doi: 10.1016/j.jointm.2021.05.002 PMC8163035 36943818

[B56] Petrey A. C. Qeadan F. Middleton E. A. Pinchuk I. V. Campbell R. A. (2021). And Beswick, E Cytokine Release Syndrome in Covid-19: Innate Immune, Vascular, and Platelet Pathogenic Factors Differ in Severity of Disease and Sex. J. Leukocyte Biol. 109, 55–66. doi: 10.1002/JLB.3COVA0820-410RRR PMC7902354 32930456

[B57] Pontecorvi G. Bellenghi M. Ortona E. Carè A. (2020). Micrornas as New Possible Actors in Gender Disparities of Covid-19 Pandemic. Acta Physiol. (Oxf. Engl.) 230, e13538. doi: 10.1111/apha.13538 PMC7404333 32672403

[B58] Roberts C. A. Lewis M. E. Boocock P. (1998). Infectious Disease, Sex, and Gender: The Complexity of it All. Sex Gender Paleopathol. Perspect. 93–113.

[B59] Sapey E. Gallier S. Mainey C. Nightingale P. McNulty D. Crothers H.. (2020). Ethnicity and Risk of Death in Patients Hospitalised for Covid-19 Infection in the Uk: An Observational Cohort Study in an Urban Catchment Area. BMJ Open Respir. Res. 7, e000644. doi: 10.1136/bmjresp-2020-000644 PMC7467523 32873607

[B60] Sayad B. Rahimi Z. (2020). Blood Coagulation Parameters in Patients With Severe Covid-19 From Kermanshah Province, Islamic Republic of Iran. Eastern Mediterranean Health J. 26, 999–1004. doi: 10.26719/emhj.20.105 33047789

[B61] Schlagenhauf P. Chen L. H. Wilson M. E. Freedman D. O. Tcheng D. Schwartz E.. (2010). Sex and Gender Differences in Travel-Associated Disease. Clin. Infect. Dis. 50, 826–832. doi: 10.1086/650575 20156059

[B62] Sha J. Qie G. Yao Q. Sun W. Wang C. Zhang Z.. (2021). Sex Differences on Clinical Characteristics, Severity, and Mortality in Adult Patients With Covid-19: A Multicentre Retrospective Study. Front. Med. 8, 123. doi: 10.3389/fmed.2021.607059 PMC7906985 33644092

[B63] Shi H. Han X. Jiang N. Cao Y. Alwalid O. Gu J.. (2020). Radiological Findings From81 Patients With Covid-19 Pneumonia in Wuhan, China: A Descriptive Study. Lancet Infect. Dis. 20, 425–434. doi: 10.1016/S1473-3099(20)30086-4 32105637 PMC7159053

[B64] Statsenko Y. Al Zahmi F. Habuza T. Neidl-Van Gorkom K. Zaki N. (2021). Prediction of Covid-19 Severity Using Laboratory Findings on Admission: Informative Values, Thresholds, Ml Model Performance. BMJ Open 11, e044500. doi: 10.1136/bmjopen-2020-044500 PMC7918887 33637550

[B65] Takahashi T. Ellingson M. K. Wong P. Israelow B. Lucas C. Klein J.. (2020). Sex Differences in Immune Responses That Underlie Covid-19 Disease Outcomes. Nature 588, 315–320. doi: 10.1038/s41586-020-2700-3 32846427 PMC7725931

[B66] Tolhurst R. De Koning K. Price J. Kemp J. Theobald S. Squire S. (2002). The Challenge of Infectious Disease: Time to Take Gender Into Account. J. Health Manage. 4, 135–151. doi: 10.1177/097206340200400204

[B67] Toussie D. Voutsinas N. Finkelstein M. Cedillo M. A. Manna S. Maron S. Z.. (2020). Clinical and Chest Radiography Features Determine Patient Outcomes in Young and Middle-Aged Adults With Covid-19. Radiology 297, E197–E206. doi: 10.1148/radiol.2020201754 32407255 PMC7507999

[B68] Wang M. Jiang N. Li C. Wang J. Yang H. Liu L.. (2021). Sex-Disaggregated Data on Clinical Characteristics and Outcomes of Hospitalized Patients With Covid-19: A Retrospective Study. Front. Cell. Infect. Microbiol. 11, 467. doi: 10.3389/fcimb.2021.680422 PMC8187910 34123876

[B69] Wang J. Zhu X. Xu Z. Yang G. Mao G. Jia Y.. (2020). Clinical and Ct Findings of Covid-19:Differences Among Three Age Groups. BMC Infect. Dis. 20, 1–11. doi: 10.1186/s12879-020-05154-9 PMC7306933 32571228

[B70] Whitacre C. C. Reingold S. C. O’Looney P. A. Blankenhorn E. Brinley F. Collier E.. (1999). A Gender Gap in Autoimmunity: Task Force on Gender, Multiple Sclerosis and Autoimmunity. Science 283, 1277–1278. doi: 10.1126/science.283.5406.1277 10084932

[B71] Williamson E. J. Walker A. J. Bhaskaran K. Bacon S. Bates C. Morton C. E.. (2020). Factors Associated With Covid-19-Related Death Using Opensafely. Nature 584, 430–436. doi: 10.1038/s41586-020-2521-4 32640463 PMC7611074

[B72] Wray S. Arrowsmith S. (2021). The Physiological Mechanisms of the Sex-Based Difference in Outcomes of Covid19 Infection. Front. Physiol. 12, 71. doi: 10.3389/fphys.2021.627260 PMC7900431 33633588

[B73] Xie J. Covassin N. Fan Z. Singh P. Gao W. Li G.. (2020). Association Between Hypoxemia and Mortality in Patients With Covid-19. In Mayo Clin. Proc. (Elsevier) 95, 1138–1147. doi: 10.1016/j.mayocp.2020.04.006 PMC7151468 32376101

[B74] Xu K. Wei Y. Giunta S. Zhou M. Xia S. (2021). Do Inflammaging and Coagul-Aging Play a Role as Conditions Contributing to the Co-Occurrence of the Severe Hyper-Inflammatory State and Deadly Coagulopathy During Covid-19 in Older People? Exp. Gerontol. 151, 111423. doi: 10.1016/j.exger.2021.111423 34048906 PMC8149167

[B75] Yang X. Yu Y. Xu J. Shu H. Liu H. Wu Y.. (2020). Clinical Course and Outcomes of Critically Ill Patients With Sars-Cov-2 Pneumonia in Wuhan, China: A Single-Centered, Retrospective, Observational Study. Lancet Respir. Med. 8, 475–481. doi: 10.1016/S2213-2600(20)30079-5 32105632 PMC7102538

[B76] Zhao M. Wang M. Zhang J. Gu J. Zhang P. Xu Y.. (2020). Comparison of Clinical Characteristics and Outcomes of Patients With Coronavirus Disease 2019 at Different Ages. Aging (Albany NY) 12, 10070. doi: 10.18632/aging.103298 32499448 PMC7346026

[B77] Zhou F. Yu T. Du R. Fan G. Liu Y. Liu Z.. (2020). Clinical Course and Risk Factors for Mortality of Adult Inpatients With Covid-19 in Wuhan, China: A Retrospective Cohort Study. Lancet 395, 1054–1062. doi: 10.1016/S0140-6736(20)30566-3 32171076 PMC7270627

[B78] Zhu T. Wang Y. Zhou S. Zhang N. Xia L. (2020). A Comparative Study of Chest Computed Tomography Features in Young and Older Adults With Corona Virus Disease (Covid-19). J. Thorac. Imaging 35, W97. doi: 10.1097/RTI.0000000000000513 32235187 PMC7253040

