# Supplementary material for: Impact of Age and Sex on COVID-19 Severity Assessed From Radiologic and Clinical Findings
Source: Front Cell Infect Microbiol. 2022 Feb 25;11:777070. doi: 10.3389/fcimb.2021.777070 (PMC8913498; doi:10.3389/fcimb.2021.777070)
Supplement: Supplementary file 1 [file DataSheet_1.pdf]

Supplemental Table 1. Comparison of subjects with regards to age groups and gender, Abu Dhabi Emirate

		Both sexes				All ages			18-39 years			40-64 years			≥65 years		
	**Total** n=605	**18-39** n_1=341(56.36%)	**40-64** n_2=253(41.82%)	**≥65** n_3=11(1.82%)	p_{1-3}	**Female** n_4=86(14.21%)	**Male** n_5=519(85.79%)	p_{4-5}	**Female** n_6=47(13.78%)	**Male** n_7=294(86.22%)	p_{6-7}	**Female** n_8=33(13.04%)	**Male** n_9=220(86.96%)	p_{8-9}	**Female** n_{10}=6(54.55%)	**Male** n_{11}=5(45.45%)	p_{10-11}
Age	39.78[31.26-47.38]	31.57±5.26*	49.33±6.36*	74.79±8.38*	**<0.001**	40.91±14.09	39.6±11.0	0.4515	30.9±5.18	31.68±5.26	0.1677	48.83±6.42	49.41±6.35	0.2740	75.76±8.13	73.64±8.52	0.4271
BMI	28.02[23.76-28.78]	27.57±19.04*	28.65±21.61*	27.36±5.68	**0.0076**	27.18±5.54	28.15±21.42	0.3379	25.76±4.95	27.84±20.29	0.1147	28.61±5.56	28.66±23.08	*0.0530*	29.54±6.48	24.74±2.84	0.2057
Temperature, $^\circ C$	36.82[36.6-37.0]	36.79±0.37	36.86±0.39	36.73±0.29	0.1484	36.81±0.36	36.82±0.38	0.4743	36.76±0.26	36.79±0.39	0.4820	36.92±0.45	36.85±0.38	0.1704	36.58±0.3	36.9±0.13	**0.0578**
SBP	135.83[124.0-150.0]	134.63±20.31*	137.78±26.87*	128.34±34.82	**0.0118**	129.4±21.68	136.9±23.82	**0.0001**	123.86±21.05	136.34±19.65	**<0.001**	135.76±16.81	138.09±28.07	0.0914	136.97±34.57	117.98±32.19	0.2057
DBP	81.17[73.0-92.0]	81.33±14.94*	81.98±20.41*	57.8±21.94*	**0.0002**	78.42±16.23	81.62±18.09	**0.0227**	77.2±15.09	81.98±14.81	**0.0335**	84.09±13.39	81.66±21.25	0.4070	56.67±18.48	59.16±25.41	0.4636
**OXYGENATION**																	
SpO2	0.99[0.98-1.0]	0.99±0.01*	0.98±0.03*	0.96±0.04*	**<0.001**	0.99±0.01	0.99±0.02	0.0932	0.99±0.01	0.99±0.01	**0.0497**	0.99±0.01	0.98±0.03	0.2823	0.97±0.03	0.95±0.04	0.3883
Heart beat rate	85.47[76.0-93.0]	84.92±14.72	86.17±13.97	86.82±10.94	0.3793	88.33±13.85	85.0±14.39	**0.0237**	86.13±14.34	84.72±14.78	0.3068	91.72±12.55	85.34±13.98	**0.0047**	87.17±13.07	86.4±7.61	0.2911
Breath rate	18.26[18.0-18.0]	17.97±1.6*	18.51±2.85*	21.55±5.05*	**<0.001**	18.15±1.69	18.28±2.45	0.4910	17.91±1.63	17.98±1.59	0.3369	18.18±1.49	18.56±3.0	0.3810	19.83±2.19	23.6±6.53	0.3161
Anion gap	17.31[16.5-18.3]	17.3±2.73	17.39±2.44	15.29±3.47*	0.0663	17.2±2.1	17.32±2.72	0.2421	17.27±1.8	17.31±2.85	0.2102	17.7±1.43	17.34±2.55	0.1329	13.87±3.78	17.42±1.03	**0.0211**
Increased anion gap	503(83.14%)	285(83.58%)	213(84.19%)	5(45.45%)*	**0.0008**	72(83.72%)	431(83.04%)	0.8036	39(82.98%)	246(83.67%)	0.9098	31(93.94%)	182(82.73%)	0.2520	2(33.33%)	3(60.0%)	na
**BIOCHEMICAL DATA ON ADMISSION**																	
**Electrolytes**																	
- K^+	4.03[3.8-4.3]	4.0±0.35*	4.08±0.4*	4.01±0.38	**0.0376**	3.92±0.36	4.05±0.37	**0.0021**	3.86±0.34	4.02±0.35	**0.0046**	3.98±0.37	4.09±0.4	0.0604	4.03±0.41	3.97±0.33	0.4576
- Na^+	139.36[138.0-141.0]	139.7±2.13*	138.91±3.03*	139.5±2.42	**0.0090**	139.05±2.42	139.42±2.6	0.0997	139.06±2.03	139.8±2.13	**0.0176**	139.0±2.9	138.9±3.05	0.3659	139.17±2.27	140.0±2.55	0.4151
- HCO_3^-	25.04[23.7-26.3]	25.14±3.08	24.82±2.7	26.99±5.26	0.2087	24.07±2.88	25.2±2.97	**<0.001**	23.47±2.06	25.41±3.13	**<0.001**	24.03±2.11	24.94±2.75	**0.0228**	28.97±5.87	24.02±1.63	0.0829
- Cl^-	101.05[99.4-102.9]	101.24±2.49	100.78±3.18	101.23±3.38	0.3572	101.7±2.99	100.94±2.78	**0.0034**	102.18±3.13	101.09±2.34	**0.0006**	101.26±2.52	100.71±3.26	0.1915	100.37±3.46	102.52±2.8	0.1687
- Ca^{2+}	2.35[2.29-2.42]	2.36±0.1*	2.33±0.11*	2.32±0.1	**0.0002**	2.33±0.1	2.35±0.1	*0.0538*	2.35±0.1	2.37±0.1	0.3943	2.29±0.08	2.34±0.11	**0.0054**	2.38±0.05	2.24±0.1	0.1080
- Mg^{2+}	0.84[0.79-0.89]	0.84±0.07	0.85±0.08	0.77±0.03*	**0.0029**	0.81±0.07	0.85±0.08	**0.0002**	0.8±0.07	0.85±0.07	**0.0006**	0.83±0.08	0.85±0.08	0.0951	0.79±0.02	0.74±0.01	**0.0351**
- *Phosphate*	1.12[0.95-1.27]	1.14±0.23*	1.1±0.23	1.0±0.2	*0.0571*	1.08±0.17	1.12±0.24	0.0862	1.1±0.14	1.14±0.25	0.1149	1.06±0.2	1.1±0.23	0.1890	1.09±0.17	0.88±0.18	0.0890
**Substrates**																	
CRP	9.89[0.8-6.3]	6.51±18.47*	13.48±28.26*	32.36±47.17	**<0.001**	17.1±33.58	8.71±22.14	**0.0264**	12.9±31.79	5.53±15.2	0.3173	21.23±33.6	12.31±27.17	**0.0182**	25.95±40.98	40.06±52.63	0.4635
D-Dimer	0.37[0.19-0.37]	0.34±0.42*	0.41±0.41*	0.6±0.47	**<0.001**	0.57±0.76	0.34±0.32	**<0.001**	0.65±0.93	0.29±0.22	**<0.001**	0.47±0.46	0.4±0.41	**0.0393**	0.45±0.36	0.8±0.51	0.1059
Ferritin	360.79[127.75-411.25]	282.4±334.96*	456.49±538.78*	756.38±1150.01	**<0.001**	169.0±433.04	391.32±457.43	**<0.001**	87.4±73.2	312.03±348.96	**<0.001**	280.87±663.04	483.31±511.91	**<0.001**	179.25±152.93	1333.5±1398.4	**0.0303**
Total protein	78.08[75.0-82.0]	78.67±5.51*	77.32±4.82*	75.33±2.05	**0.0132**	77.82±5.64	78.13±5.2	0.4778	77.61±6.53	78.84±5.3	0.2284	78.36±4.12	77.16±4.9	0.0991	75.5±2.5	75.0±0.0	0.2701
Albumin	39.94[38.0-43.0]	41.25±3.92*	38.36±4.83*	35.14±5.46*	**<0.001**	38.39±4.7	40.2±4.54	**0.0002**	39.05±5.2	41.63±3.51	**0.0003**	37.61±3.88	38.47±4.95	*0.0810*	36.75±2.59	33.0±7.26	0.4286
Urea	4.09[3.0-4.6]	3.73±1.1*	4.46±2.68*	6.95±3.51*	**<0.001**	3.38±1.33	4.21±2.11	**<0.001**	3.04±0.93	3.84±1.08	**<0.001**	3.42±0.93	4.62±2.82	**0.0002**	5.92±2.58	8.5±4.09	0.1679
Creatinine	81.11[66.0-87.0]	76.73±21.76	87.2±74.49	78.2±34.75	0.1688	56.71±12.08	85.18±54.05	**<0.001**	56.26±10.69	80.0±21.29	**<0.001**	56.24±10.2	91.9±78.8	**<0.001**	62.83±24.13	101.25±35.49	*0.0549*
Uric acid	306.38[253.0-353.0]	311.87±77.43*	298.71±89.69*	307.0±117.69	**0.0419**	263.05±84.53	313.45±81.32	**<0.001**	253.16±61.87	321.15±75.55	**<0.001**	264.83±95.21	303.9±87.66	**0.0050**	356.0±136.76	258.0±64.8	0.2352
Total bilirubin, $umol/L$	8.99[5.7-10.4]	8.81±4.96	9.26±7.02	7.64±3.88	0.4129	6.44±3.9	9.39±6.05	**<0.001**	6.03±3.49	9.26±5.02	**<0.001**	7.18±4.5	9.56±7.26	**0.0006**	5.25±1.15	9.23±4.23	0.3864
Direct bilirubin, $umol/L$	3.54[2.3-3.9]	3.3±1.56	3.89±5.6	3.62±2.48	0.2393	2.9±2.46	3.65±3.96	**<0.001**	2.6±1.35	3.41±1.57	**<0.001**	3.44±3.58	3.95±5.83	**0.0012**	2.1±0.2	5.15±2.75	0.1226
Glucose random	6.41[5.05-6.54]	5.77±1.65*	7.31±3.19*	6.8±1.97	**<0.001**	6.57±2.79	6.38±2.47	0.1449	5.97±2.12	5.74±1.56	0.1568	7.38±3.45	7.3±3.14	0.3467	7.24±2.26	5.92±0.53	0.4085
**Enzymes**																	
G6PDH	10.9[9.9-12.4]	10.89±3.42	10.93±2.84	9.9±7.1	0.7419	12.46±3.17	10.59±3.21	**0.0002**	12.69±3.21	10.54±3.35	**0.0014**	11.75±3.02	10.79±2.78	0.0919	14.8±1.9	0.1±0.0	0.2701
LDH	227.02[181.0-244.0]	215.35±62.39*	241.63±99.37*	263.29±145.39	**0.0030**	220.99±62.92	227.95±85.13	0.3504	201.83±38.62	217.45±65.05	0.1592	254.96±79.93	239.83±101.58	0.1036	188.0±14.61	363.67±177.21	0.2979
Alkaline phosphatase	80.64[63.25-86.0]	82.29±57.01	78.38±31.12	82.6±41.56	0.6253	80.38±38.98	80.69±48.95	0.4212	82.19±47.71	82.3±58.41	0.1497	79.75±19.56	78.18±32.42	0.0970	54.5±20.5	101.33±41.48	0.3835
Amylase	79.83[55.0-90.0]	78.54±61.96	80.37±40.22	115.88±78.88	0.4678	69.47±27.62	81.55±57.75	**0.0195**	71.3±29.16	79.71±65.65	0.1930	65.4±24.69	82.68±41.63	**0.0104**	80.0±26.11	151.75±95.85	0.2352
Lipase	41.26[25.0-45.0]	34.95±15.93*	49.6±60.36*	59.0±80.43	**0.0001**	33.13±12.35	42.61±45.1	**0.0189**	33.02±12.23	35.26±16.43	0.2946	33.6±13.0	52.06±64.28	**0.0073**	30.75±7.29	96.67±111.98	0.2979
**HAEMATOLOGIC DATA ON ADMISSION**																	
Platelet	260.17[208.0-302.0]	259.75±67.55	258.33±83.58	313.09±153.88	0.4076	283.41±92.5	256.43±73.92	**0.0060**	280.57±84.21	256.57±64.03	0.0708	266.45±67.5	257.14±85.62	0.0950	391.83±163.25	218.6±61.19	**0.0276**
**Leucocytes**																	
WBC	6.87[5.3-8.1]	6.9±2.28	6.82±1.98	6.99±1.3	0.8866	7.14±2.81	6.83±2.03	0.3588	7.84±3.16	6.77±2.09	**0.0450**	6.12±2.14	6.92±1.93	**0.0397**	7.3±1.08	6.47±1.46	0.2755
Lymphocytes, abs	2.17[1.58-2.68]	2.26±0.81*	2.05±0.81*	1.77±0.6	**0.0012**	2.15±0.75	2.17±0.82	0.4222	2.32±0.71	2.25±0.82	0.1492	1.95±0.76	2.07±0.81	0.2428	1.91±0.67	1.61±0.46	0.3240
Lymphocytes, %	32.42[24.48-39.3]	33.92±10.53*	30.66±10.11*	26.11±13.53*	**0.0002**	32.3±12.42	32.44±10.25	0.3734	32.75±11.95	34.1±10.29	0.2185	32.55±12.36	30.38±9.7	0.1880	27.67±14.96	24.24±11.29	0.4636
Baso, abs	0.03[0.02-0.04]	0.03±0.02	0.03±0.02	0.04±0.03	0.6136	0.03±0.02	0.03±0.02	0.2184	0.03±0.02	0.03±0.02	0.1829	0.03±0.02	0.03±0.02	0.2879	0.05±0.03	0.02±0.01	*0.0514*
Baso, %	0.43[0.3-0.6]	0.42±0.23	0.44±0.26	0.48±0.36	0.6938	0.45±0.25	0.43±0.24	0.2484	0.41±0.19	0.42±0.24	0.4946	0.45±0.26	0.44±0.25	0.3854	0.67±0.39	0.26±0.08	**0.0071**
Eos, abs	0.17[0.05-0.21]	0.18±0.24	0.17±0.23	0.17±0.15	0.8590	0.11±0.12	0.18±0.25	**0.0060**	0.12±0.12	0.19±0.25	*0.0561*	0.1±0.12	0.18±0.24	**0.0137**	0.13±0.07	0.21±0.19	0.4274
Eos, %	2.43[0.8-3.0]	2.51±3.24	2.32±2.68	2.35±2.09	0.8266	1.63±1.51	2.56±3.16	**0.0049**	1.65±1.51	2.64±3.41	**0.0261**	1.56±1.56	2.43±2.78	**0.0319**	1.85±1.15	2.94±2.71	0.4274
Mono, abs	0.59[0.44-0.71]	0.58±0.23	0.59±0.21	0.79±0.28*	**0.0290**	0.58±0.3	0.59±0.21	0.1214	0.62±0.31	0.58±0.21	0.3715	0.51±0.26	0.6±0.2	**0.0035**	0.69±0.2	0.9±0.32	0.2057
Mono, %	8.77[6.7-10.2]	8.7±2.88	8.81±2.87	10.24±2.81	0.2134	8.16±2.86	8.87±2.88	**0.0080**	8.09±2.54	8.79±2.92	0.1002	7.99±3.21	8.93±2.8	**0.0074**	9.55±2.76	11.06±2.66	0.3240
Neutro, abs	3.99[2.75-4.77]	3.88±1.84*	4.07±1.69	5.78±5.5	0.0705	4.37±2.42	3.93±1.84	0.1259	4.63±2.5	3.76±1.69	**0.0209**	3.97±2.4	4.09±1.56	0.1292	4.6±1.52	7.21±7.75	0.2614
Neutro, %	55.94[47.9-64.9]	54.43±11.95*	57.77±11.41*	60.83±15.75	**0.0022**	57.47±13.77	55.69±11.61	0.0686	57.1±13.0	54.03±11.72	*0.0572*	57.45±14.21	57.82±10.95	0.4919	60.27±16.4	61.5±14.89	0.2057
**Red blood cells**																	
Hgb, g/l	144.04[135.0-154.75]	147.54±14.94*	140.65±16.94*	113.18±16.47*	**<0.001**	124.15±15.02	147.2±14.69	**<0.001**	125.48±16.12	150.89±11.52	**<0.001**	124.74±11.96	142.95±16.31	**<0.001**	111.33±14.88	115.4±17.94	0.3237
Hct, %	0.43[0.4-0.45]	0.44±0.04*	0.42±0.05*	0.35±0.06*	**<0.001**	0.38±0.04	0.43±0.04	**<0.001**	0.38±0.04	0.44±0.03	**<0.001**	0.38±0.03	0.42±0.04	**<0.001**	0.35±0.06	0.35±0.06	0.4274
RBC,	4.5[4.56-5.4]	4.77±1.58*	4.21±1.89*	2.92±1.83*	**<0.001**	3.86±1.6	4.61±1.76	**<0.001**	3.88±1.66	4.9±1.52	**<0.001**	3.94±1.48	4.25±1.94	**<0.001**	3.28±1.61	2.48±1.98	0.3572
MCH	338.14[330.0-346.0]	338.8±12.7	337.55±11.96	331.45±11.95*	**0.0416**	330.95±13.14	339.29±11.92	**<0.001**	331.7±14.62	339.88±12.02	**0.0001**	330.55±11.39	338.57±11.7	**0.0001**	327.5±8.67	336.2±13.51	0.2915
MCHC	338.14[330.0-346.0]	338.8±12.7	337.55±11.96	331.45±11.95*	**0.0416**	330.95±13.14	339.29±11.92	**<0.001**	331.7±14.62	339.88±12.02	**0.0001**	330.55±11.39	338.57±11.7	**0.0001**	327.5±8.67	336.2±13.51	0.2915
MCV	83.46[81.1-86.7]	83.21±5.48	83.7±5.61	85.51±7.09	0.1506	83.45±7.5	83.46±5.21	0.1092	82.78±7.9	83.28±5.01	0.3003	83.99±6.64	83.66±5.44	0.1274	85.57±8.06	85.44±5.72	0.3921
MPV	10.46[9.8-11.1]	10.48±0.9	10.4±0.96	11.1±1.09	0.0762	10.69±1.09	10.42±0.9	**0.0473**	10.81±1.06	10.43±0.87	**0.0181**	10.46±1.09	10.39±0.94	0.4865	10.9±1.15	11.34±0.94	0.3240
RDW_CV	1.85[0.12-0.13]	1.4±4.19*	2.19±5.41*	7.71±8.34*	**<0.001**	2.92±6.49	1.68±4.59	**0.0008**	2.18±5.89	1.28±3.85	**0.0173**	3.0±6.56	2.08±5.21	**0.0441**	8.02±7.89	7.34±8.84	0.3921
Imm_Retic_Frac	9.44[6.0-12.0]	9.1±4.9	9.75±5.86	14.0±10.77	0.3021	10.24±6.37	9.3±5.29	0.1446	9.51±5.76	9.03±4.74	0.4702	11.1±7.21	9.54±5.59	0.1076	11.5±4.5	17.33±14.97	0.4298
Retic_Cnt_Auto_n	61.2[39.15-76.0]	62.29±27.6	59.47±30.22	68.36±32.71	0.1999	57.19±26.14	61.85±29.2	0.1303	56.86±22.42	63.15±28.24	0.1263	55.73±30.86	60.05±30.08	0.1616	71.22±19.25	64.53±44.47	0.2979
Retic_Cnt_Auto_p	0.13[0.01-0.02]	0.1±0.45	0.16±0.67	0.55±1.33	0.3408	0.16±0.6	0.13±0.57	**0.0106**	0.1±0.42	0.1±0.45	0.1091	0.27±0.81	0.14±0.65	*0.0489*	0.02±0.0	1.27±1.79	0.2979

Supplemental Table 2. Comparison of clinical findings in patients with regards to age group and gender, Dubai Emirate

	Total	Both sexes				All ages			18-39 years			40-64 years			≥65 years		
	**Total** n=560	**18-39** n_1=292(52.14%)	**40-64** n_2=236(42.14%)	**≥65** n_3=32(5.71%)	p_{1-3}	**Female** n_4=189(33.75%)	**Male** n_5=371(66.25%)	p_{4-5}	**Female** n_6=119(40.75%)	**Male** n_7=173(59.25%)	p_{6-7}	**Female** n_8=55(23.31%)	**Male** n_9=181(76.69%)	p_{8-9}	**Female** n_{10}=15(46.88%)	**Male** n_{11}=17(53.12%)	p_{10-11}
Age	39.0[33.0-49.0]	33.0±4.8*	47.0±7.16*	70.0±7.06*	**<0.001**	36.0±13.17	41.0±12.26	**<0.0027**	33.0±4.57	33.0±4.95	0.3532	47.0±8.1	47.0±6.85	0.4546	70.0±3.77	69.0±8.89	0.2781
BMI	27.02[23.88-30.44]	26.03±5.72*	27.73±4.57*	31.0±4.5*	**<0.0001**	25.29±5.9	27.29±4.94	**<0.0015**	24.0±5.83	27.07±5.43	**<0.0013**	27.62±5.02	27.76±4.35	0.2615	33.2±3.28	27.96±3.11	*<0.0179*
Temperature, °C	37.0[37.0-37.9]	37.0±0.6*	37.0±0.81*	37.0±0.63	**<0.0024**	37.0±0.6	37.0±0.75	**<0.0092**	37.0±0.47	37.0±0.67	**<0.0211**	37.0±0.76	37.0±0.82	0.3187	37.0±0.61	37.0±0.65	0.3713
SBP	124.0[114.0-135.0]	120.0±13.6*	127.5±18.84*	128.5±16.72*	**<0.001**	119.0±18.05	126.0±15.54	**<0.001**	117.0±13.58	124.0±13.1	**<0.0001**	120.0±23.87	129.0±16.89	**<0.0134**	131.0±16.22	124.0±17.08	0.4029
DBP	78.0[70.0-84.0]	76.0±9.55*	78.0±11.68*	70.0±11.3*	**<0.001**	75.0±10.92	78.0±10.67	**<0.0012**	75.0±9.47	78.0±9.48	**<0.0179**	78.0±13.39	78.0±11.04	0.1019	75.0±9.85	70.0±12.41	0.3179
**OXYGENATION**																	
Heart rate, BPM	85.0[78.0-95.0]	85.0±12.24	86.0±14.36*	80.5±17.48*	**<0.0192**	84.0±11.65	85.0±14.56	0.2453	84.0±11.89	86.0±12.42	0.1523	87.0±10.53	85.0±15.34	0.3094	81.0±12.3	80.0±20.29	0.2361
Breath rate, /min	18.0[18.0-18.0]	18.0±2.22*	18.0±4.81*	18.0±5.04*	**<0.001**	18.0±2.57	18.0±4.26	**<0.0016**	18.0±0.89	18.0±2.76	**<0.022**	18.0±2.03	18.0±5.28	**<0.0017**	22.0±6.29	18.0±2.22	**<0.0129**
SOFA score	0.0[0.0-0.0]	0.0±0.74*	0.0±2.14*	1.0±2.54*	**<0.001**	0.0±1.02	0.0±1.93	**<0.0001**	0.0±0.2	0.0±0.94	**<0.0016**	0.0±1.15	0.0±2.32	**<0.0046**	2.0±2.08	1.0±2.88	0.1513
**COMORBIDITIES**																	
Current smoking	36(6.43%)	24(8.22%)	10(4.24%)	2(6.25%)	0.1789	5(2.65%)	31(8.36%)	**<0.0098**	2(1.68%)	22(12.72%)	**<0.0004**	2(3.64%)	8(4.42%)	1	1(6.67%)	1(5.88%)	na
Chronic Cardiac disease	20(3.57%)	2(0.68%)*	6(2.54%)	12(37.5%)*	**<0.001**	5(2.65%)	15(4.04%)	0.4774	2(1.68%)		na	1(1.82%)	5(2.76%)	na	2(13.33%)	10(58.82%)	**<0.0118**
Hypertension	115(20.54%)	13(4.45%)*	75(31.78%)*	27(84.38%)*	**<0.001**	36(19.05%)	79(21.29%)	0.581	2(1.68%)	11(6.36%)	0.0814	21(38.18%)	54(29.83%)	0.2514	13(86.67%)	14(82.35%)	1
Asthma	38(6.79%)	21(7.19%)	12(5.08%)	5(15.62%)	0.0778	23(12.17%)	15(4.04%)	**<0.0006**	13(10.92%)	8(4.62%)	0.0629	6(10.91%)	6(3.31%)	**<0.0357**	4(26.67%)	1(5.88%)	na
Diabetes	98(17.5%)	11(3.77%)*	63(26.69%)*	24(75.0%)*	**<0.001**	28(14.81%)	70(18.87%)	0.2424	3(2.52%)	8(4.62%)	0.5341	14(25.45%)	49(27.07%)	0.8635	11(73.33%)	13(76.47%)	1
**DISEASE COURSE AND SEVERITY**																	
**Clinical severity**					**<0.001**			**<0.029**			**<0.0158**			0.328			0.169
- *Asymp/Mild/Moderate*	431(76.96%)	265(90.75%)*	154(65.25%)*	12(37.5%)*		158(83.6%)*	273(73.58%)*		115(96.64%)*	150(86.71%)*		39(70.91%)	115(63.54%)		4(26.67%)	8(47.06%)	
- *Severe*	83(14.82%)	20(6.85%)*	51(21.61%)*	12(37.5%)*		20(10.58%)*	63(16.98%)*		3(2.52%)*	17(9.83%)*		12(21.82%)	39(21.55%)		5(33.33%)	7(41.18%)	
- *Critical*	46(8.21%)	7(2.4%)*	31(13.14%)*	8(25.0%)*		11(5.82%)	35(9.43%)		1(0.84%)	6(3.47%)		4(7.27%)	27(14.92%)		6(40.0%)	2(11.76%)	
Need O_2 supplementation at adm	82(14.64%)	15(5.14%)*	53(22.46%)*	14(43.75%)*	**<0.001**	16(8.47%)	66(17.79%)	**<0.0034**	1(0.84%)	14(8.09%)	**<0.0055**	6(10.91%)	47(25.97%)	**<0.0257**	9(60.0%)	5(29.41%)	0.1527
Need O_2 supplementation later	112(20.0%)	25(8.56%)*	70(29.66%)*	17(53.12%)*	**<0.001**	26(13.76%)	86(23.18%)	**<0.0099**	3(2.52%)	22(12.72%)	**<0.0023**	12(21.82%)	58(32.04%)	0.1782	11(73.33%)	6(35.29%)	**<0.0416**
Admitted directly to ICU	56(10.0%)	10(3.42%)*	38(16.1%)*	8(25.0%)*	**<0.001**	9(4.76%)	47(12.67%)	**<0.0027**	1(0.84%)	9(5.2%)	*0.0521*	3(5.45%)	35(19.34%)	**<0.012**	5(33.33%)	3(17.65%)	na
Transferred to ICU later	72(12.86%)	14(4.79%)*	47(19.92%)*	11(34.38%)*	**<0.001**	14(7.41%)	58(15.63%)	**<0.0072**	2(1.68%)	12(6.94%)	**<0.0499**	4(7.27%)	43(23.76%)	**<0.0065**	8(53.33%)	3(17.65%)	0.0617
Duration of ICU stay	0.0[0.0-0.0]	0.0±1.96*	0.0±7.6*	0.0±9.67*	**<0.001**	0.0±2.57	0.0±6.81	**<0.0024**	0.0±0.78	0.0±2.44	**<0.019**	0.0±2.68	0.0±8.46	**<0.0036**	2.0±5.91	0.0±12.04	**<0.0473**
Onset to hospitalization, *days*	14.0[8.0-19.0]	11.0±5.87*	16.0±12.08*	23.0±12.79*	**<0.001**	13.0±6.75	14.0±11.62	0.1122	12.0±5.25	9.0±6.25	**<0.0102**	14.0±8.49	17.0±12.82	**<0.0075**	19.0±6.4	25.0±14.64	**<0.028**
Onset to positive PCR, *days*	2.0[1.0-5.0]	2.0±2.74*	3.0±4.65*	4.0±7.62*	**<0.0002**	2.0±3.2	2.0±4.59	0.3964	2.0±2.77	2.0±2.71	0.3105	3.0±3.6	3.0±4.9	0.429	3.5±4.07	6.0±9.52	0.0916
Duration of viral shedding	10.0[6.0-14.0]	10.0±5.38	10.0±6.97	11.0±5.4	0.6554	10.0±5.31	10.0±6.47	0.1331	10.0±5.16	10.0±5.52	0.4275	8.0±5.73	10.0±7.26	0.0574	11.0±4.52	12.5±5.71	0.1241
Length of disease, *days*	14.0[10.0-18.0]	13.0±5.4*	14.0±8.02	18.0±4.84*	**<0.0009**	13.0±5.51	14.0±7.36	0.0635	13.0±5.37	13.0±5.42	0.3403	12.0±5.88	15.0±8.45	**<0.0259**	15.0±4.59	20.5±4.24	**<0.0467**
**Outcome**																	
- *alive*	545(97.32%)	292(100.0%)*	226(95.76%)	27(84.38%)*	**<0.001**	183(96.83%)	362(97.57%)	0.5904				54(98.18%)	172(95.03%)	0.4601	10(66.67%)	17(100.0%)	**<0.0149**
- *deceased*	15(2.68%)	0(0.0%)*	10(4.24%)	5(15.62%)*		6(3.17%)	9(2.43%)					1(1.82%)	9(4.97%)		5(33.33%)*	0(0.0%)*	
**COMPLICATIONS**																	
- *Any* complications	123(21.96%)	33(11.3%)*	73(30.93%)*	17(53.12%)*	**<0.001**	24(12.7%)	99(26.68%)	**<0.0001**	5(4.2%)	28(16.18%)	**<0.0012**	9(16.36%)	64(35.36%)	**<0.0076**	10(66.67%)	7(41.18%)	0.1777
- *Count*	0.0[0.0-0.0]	0.0±0.65*	0.0±2.32*	1.0±2.98*	**<0.001**	0.0±1.35	0.0±2.0	**<0.0001**	0.0±0.33	0.0±0.79	**<0.0007**	0.0±1.27	0.0±2.52	**<0.003**	1.0±3.03	0.0±2.81	0.072
ARDS	76(13.57%)	14(4.79%)*	51(21.61%)*	11(34.38%)*	**<0.001**	16(8.47%)	60(16.17%)	**<0.0128**	2(1.68%)	12(6.94%)	**<0.0499**	6(10.91%)	45(24.86%)	**<0.0383**	8(53.33%)	3(17.65%)	0.0617
Bacterial pneumonia	15(2.68%)	2(0.68%)*	11(4.66%)*	2(6.25%)	**<0.0083**		15(4.04%)	**<0.0037**		2(1.16%)	na		11(6.08%)	0.072		2(11.76%)	na
Liver dysfunction	54(9.64%)	22(7.53%)	30(12.71%)*	2(6.25%)	0.1073	8(4.23%)	46(12.4%)	**<0.0014**	3(2.52%)	19(10.98%)	**<0.0065**	3(5.45%)	27(14.92%)	0.0684	2(13.33%)		na
Acute renal injury	47(8.39%)	6(2.05%)*	30(12.71%)*	11(34.38%)*	**<0.001**	10(5.29%)	37(9.97%)	0.0753	1(0.84%)	5(2.89%)	na	3(5.45%)	27(14.92%)	0.0684	6(40.0%)	5(29.41%)	0.712
Septic Shock	25(4.46%)	2(0.68%)*	17(7.2%)*	6(18.75%)*	**<0.001**	5(2.65%)	20(5.39%)	0.1932		2(1.16%)	na	1(1.82%)	16(8.84%)	0.1315	4(26.67%)	2(11.76%)	na
Seizure	5(0.89%)	1(0.34%)	4(1.69%)		na	1(0.53%)	4(1.08%)	na		1(0.58%)	na	1(1.82%)	3(1.66%)	na			
**Cardiac complications**																	
Myocarditis	2(0.36%)		2(0.85%)		na		2(0.54%)	na					2(1.1%)	na			
New onset Cardiomyopathy	3(0.54%)		3(1.27%)		na		3(0.81%)	na					3(1.66%)	na			
Cardiac arrythmia	15(2.68%)	1(0.34%)*	10(4.24%)	4(12.5%)*	**<0.001**	6(3.17%)	9(2.43%)	0.5904	1(0.84%)		na	2(3.64%)	8(4.42%)	1	3(20.0%)	1(5.88%)	na
Cardiac arrest	14(2.5%)		10(4.24%)*	4(12.5%)*	**<0.001**	5(2.65%)	9(2.43%)	1				1(1.82%)	9(4.97%)	0.4601	4(26.67%)		na
GCS	15.0[15.0-15.0]	15.0±0.7*	15.0±2.17	15.0±2.97*	**<0.001**	15.0±1.38	15.0±1.81	0.4751	15.0±0.0	15.0±0.91	0.2054	15.0±1.91	15.0±2.24	0.3894	15.0±3.07	15.0±2.87	0.4449
**SYMPTOMS**																	
Cough	304(54.29%)	134(45.89%)*	155(65.68%)*	15(46.88%)	**<0.001**	96(50.79%)	208(56.06%)	0.2447	50(42.02%)	84(48.55%)	0.2843	38(69.09%)	117(64.64%)	0.6276	8(53.33%)	7(41.18%)	0.7235
Sputum	29(5.18%)	8(2.74%)*	18(7.63%)*	3(9.38%)	**<0.0228**	7(3.7%)	22(5.93%)	0.3166	2(1.68%)	6(3.47%)	na	2(3.64%)	16(8.84%)	0.2575	3(20.0%)		na
Sore throat	172(30.71%)	99(33.9%)	68(28.81%)	5(15.62%)	0.0735	76(40.21%)	96(25.88%)	**<0.0007**	49(41.18%)	50(28.9%)	**<0.0329**	23(41.82%)	45(24.86%)	**<0.0179**	4(26.67%)	1(5.88%)	na
Chest pain	45(8.04%)	20(6.85%)	24(10.17%)	1(3.12%)	0.2171	15(7.94%)	30(8.09%)	1	10(8.4%)	10(5.78%)	0.4804	4(7.27%)	20(11.05%)	0.6106	1(6.67%)		na
SOB	154(27.5%)	56(19.18%)*	84(35.59%)*	14(43.75%)*	**<0.001**	49(25.93%)	105(28.3%)	0.617	27(22.69%)	29(16.76%)	0.2278	13(23.64%)	71(39.23%)	**<0.0373**	9(60.0%)	5(29.41%)	0.1527
Fever	327(58.39%)	156(53.42%)*	155(65.68%)*	16(50.0%)	**<0.0108**	100(52.91%)	227(61.19%)	0.0698	64(53.78%)	92(53.18%)	1	30(54.55%)	125(69.06%)	0.0529	6(40.0%)	10(58.82%)	0.4795
Headache	112(20.0%)	74(25.34%)*	36(15.25%)*	2(6.25%)*	**<0.0021**	47(24.87%)	65(17.52%)	**<0.0445**	35(29.41%)	39(22.54%)	0.218	11(20.0%)	25(13.81%)	0.2863	1(6.67%)	1(5.88%)	na
confusion	5(0.89%)		3(1.27%)	2(6.25%)*	na	4(2.12%)	1(0.27%)	na				2(3.64%)	1(0.55%)	na	2(13.33%)		na
Nausea or Vomiting	25(4.46%)	11(3.77%)	14(5.93%)		0.2209	10(5.29%)	15(4.04%)	0.5201	4(3.36%)	7(4.05%)	1	6(10.91%)	8(4.42%)	0.0994			
Any gastrointestinal symptoms	91(16.25%)	49(16.78%)	37(15.68%)	5(15.62%)	0.9388	37(19.58%)	54(14.56%)	0.146	22(18.49%)	27(15.61%)	0.5277	13(23.64%)	24(13.26%)	0.0883	2(13.33%)	3(17.65%)	na
Diarrhea	76(13.57%)	42(14.38%)	29(12.29%)	5(15.62%)	0.7369	32(16.93%)	44(11.86%)	0.117	19(15.97%)	23(13.29%)	0.6111	11(20.0%)	18(9.94%)	0.06	2(13.33%)	3(17.65%)	na
Myalgia	221(39.46%)	118(40.41%)	96(40.68%)	7(21.88%)*	0.1108	83(43.92%)	138(37.2%)	0.1435	54(45.38%)	64(36.99%)	0.1819	25(45.45%)	71(39.23%)	0.4359	4(26.67%)	3(17.65%)	na
Malaise	121(21.61%)	59(20.21%)	56(23.73%)	6(18.75%)	0.5712	41(21.69%)	80(21.56%)	1	27(22.69%)	32(18.5%)	0.4586	9(16.36%)	47(25.97%)	0.1531	5(33.33%)	1(5.88%)	na
Loss of Smell or Taste	122(21.79%)	70(23.97%)	50(21.19%)	2(6.25%)*	0.0671	51(26.98%)	71(19.14%)	**<0.0396**	37(31.09%)	33(19.08%)	**<0.0252**	13(23.64%)	37(20.44%)	0.7064	1(6.67%)	1(5.88%)	na

Supplemental Table 3. Comparison of laboratory findings in patients with regards to age group and gender, Dubai Emirate

		Both sexes				All ages			18-39 years			40-64 years			≥65 years		
	**Total** n=560	**18-39** n_1=292(52.14%)	**40-64** n_2=236(42.14%)	**≥65** n_3=32(5.71%)	p_{1-3}	**Female** n_4=189(33.75%)	**Male** n_5=371(66.25%)	p_{4-5}	**Female** n_6=119(40.75%)	**Male** n_7=173(59.25%)	p_{6-7}	**Female** n_8=55(23.31%)	**Male** n_9=181(76.69%)	p_{8-9}	**Female** n_{10}=15(46.88%)	**Male** n_{11}=17(53.12%)	p_{10-11}
**BIOCHEMICAL ANALYSIS AND COAGULATION**																	
**Coagulation**																	
APTT on adm (sec)	37.4[35.0-41.0]	37.75±4.45	37.0±11.21	40.7±22.91*	**<0.001**	38.0±11.09	37.1±9.27	0.3145	38.1±4.5	37.3±4.41	0.12	36.9±5.1	37.0±12.42	0.234	39.5±32.86	41.9±5.06	0.0618
APTT peak	38.0[35.15-42.25]	38.0±13.2	37.5±23.09	44.0±33.13*	**<0.0002**	38.5±16.13	37.95±21.34	0.2863	38.6±5.83	37.5±16.32	0.1801	37.1±5.52	37.8±25.88	0.058	40.0±46.79	44.0±6.08	0.1911
**Cytokine**																	
IL-6 (pg/mL)	25.0[7.95-34.0]	18.0±7.82	26.2±51.58	5.4±0.0	0.6451	16.0±16.03	26.2±52.71	0.2436	17.0±1.0	33.5±0.0	0.2701	30.75±20.25	26.2±54.64	0.4661			
**Substrates**																	
CRP on adm (mg/L)	5.8[1.8-27.0]	3.8±37.33*	15.2±69.72*	38.5±94.68*	**<0.001**	4.3±41.28	7.5±67.63	**<0.001**	3.4±16.78	4.0±46.19	**<0.0045**	6.2±36.67	19.05±75.76	**<0.0085**	53.0±96.45	34.0±93.05	0.4104
CRP peak (mg/L)	6.5[1.9-50.0]	4.0±51.89*	23.4±90.75*	98.0±101.12*	**<0.001**	4.7±53.53	8.5±87.8	**<0.001**	3.8±30.31	4.2±62.01	<0.0038	9.0±47.21	38.7±98.26	**<0.0053**	86.0±106.0	125.0±96.6	0.44
D-Dimer on admin (ug/L)	0.4[0.2-0.6]	0.3±1.6*	0.4±1.36*	1.0±1.51*	**<0.001**	0.4±1.05	0.3±1.68	0.185	0.3±1.16	0.3±1.82	**<0.0053**	0.4±0.52	0.4±1.51	0.2849	0.8±1.42	1.1±1.58	0.1608
D-Dimer peak (ug/L)	0.4[0.3-0.7]	0.3±2.12*	0.5±4.69*	1.2±5.07*	**<0.001**	0.4±2.6	0.4±4.21	0.2727	0.3±1.16	0.3±2.53	**<0.0157**	0.45±0.7	0.5±5.25	0.1938	1.2±6.78	1.1±1.87	0.2759
Fibrinogen on adm (mg/dL)	398.0[330.0-530.5]	357.0±220.44*	467.0±171.98*	512.0±159.92*	**<0.001**	389.5±258.87	405.0±172.09	0.1125	359.0±312.59	349.0±127.63	0.1607	413.0±128.98	487.5±179.32	*0.0536*	544.0±135.73	430.5±180.9	0.4002
Fibrinogen Peak (mg/dL)	406.0[332.5-559.0]	360.0±234.2*	490.0±489.88*	566.5±157.71*	**<0.001**	390.0±262.41	413.0±416.6	0.0638	360.0±314.57	361.0±161.67	0.2727	432.0±134.29	506.5±542.06	0.0556	561.5±146.36	613.0±167.35	0.3147
Ferritin on adm (ng/mL)	222.89[85.0-475.5]	141.1±677.75*	308.0±1463.31*	317.93±2443.25*	**<0.001**	65.0±162.87	308.0±1491.51	**<0.001**	51.0±108.06	250.0±835.21	**<0.001**	85.5±188.49	379.0±1616.16	**<0.001**	291.0±205.22	326.09±3226.88	0.1631
Ferritin Peak (ng/mL)	235.0[90.38-600.0]	144.5±823.53*	354.0±5969.47*	662.5±6029.31*	**<0.001**	68.5±2564.05	352.0±4912.64	**<0.001**	52.0±162.81	250.0±1011.23	**<0.001**	102.0±357.01	428.0±6670.58	**<0.001**	658.0±8071.03	667.0±3220.08	0.44
Troponin on adm (ng/mL)	0.0[0.0-0.0]	0.0±0.19*	0.0±0.8	0.0±0.04*	**<0.001**	0.0±0.01	0.0±0.67	<0.0069	0.0±0.0	0.0±0.24	0.0806	0.0±0.0	0.0±0.91	<0.0137	0.0±0.02	0.0±0.04	0.2094
Troponin Peak ()	0.0[0.0-0.0]	0.0±0.19*	0.0±1.15*	0.0±0.15*	**<0.001**	0.0±0.07	0.0±0.94	**<0.0291**	0.0±0.0	0.0±0.25	*0.0522*	0.0±0.06	0.0±1.3	**<0.024**	0.0±0.18	0.0±0.1	0.1146
Creatinine on adm (umol/L)	76.0[67.0-89.0]	74.0±15.81*	78.0±31.99*	86.0±88.01	**<0.0008**	65.0±23.62	84.8±34.15	**<0.001**	64.9±8.24	84.0±14.08	**<0.001**	66.0±8.53	85.0±34.2	**<0.001**	71.0±70.59	90.0±100.56	**<0.0321**
Creatinine peak (umol/L)	77.85[67.78-91.0]	74.1±16.12*	81.0±57.51*	90.0±93.08*	**<0.001**	66.0±29.15	86.0±51.41	**<0.001**	64.9±8.46	84.7±14.35	**<0.001**	66.5±10.35	86.6±63.18	**<0.001**	87.0±80.26	93.0±103.07	0.305
Total Bilirubin on Adm (umol/L)	9.0[6.0-12.5]	8.2±5.43*	10.0±6.8*	9.0±4.07	<0.0006	6.0±3.83	10.0±6.38	<**0.001**	6.0±3.61	9.85±5.8	<**0.001**	6.0±4.16	10.4±6.96	<**0.001**	8.8±3.79	10.0±4.2	0.177
Total Bilirubin Peak (umol/L)	9.8[6.5-14.15]	8.7±7.96*	10.75±22.28*	10.0±7.72	**<0.001**	6.4±5.61	11.0±18.6	**<0.001**	6.1±5.94	10.1±8.57	**<0.001**	6.85±4.74	12.25±24.77	**<0.001**	10.4±4.83	10.0±9.37	0.279
**Enzymes**																	
LDH on adm (U/L)	192.0[159.0-264.0]	175.0±112.33*	226.0±195.78*	243.0±140.3*	**<0.001**	177.0±84.08	202.5±183.17	**<0.001**	165.0±49.68	182.5±134.86	**<0.001**	220.0±102.38	226.5±213.73	0.1478	237.0±109.85	255.0±163.14	0.27
LDH peak (U/L)	195.0[160.0-280.0]	178.0±143.48*	227.0±622.28*	253.0±1305.62*	**<0.001**	180.0±615.08	204.5±513.58	**<0.0001**	168.0±63.18	182.5±172.84	**<0.0001**	226.0±169.08	228.5±697.35	0.1843	251.0±1815.24	255.0±164.76	0.3106
ALT on adm (U/L)	28.5[18.0-48.75]	25.0±39.49*	31.0±32.42*	24.0±16.71	**<0.0014**	17.0±26.69	35.0±37.83	**<0.001**	16.0±26.51	36.0±43.59	**<0.001**	22.5±28.55	35.0±32.81	**<0.001**	23.0±16.53	34.0±16.4	0.1771
ALT peak (U/L)	32.0[19.0-68.0]	26.0±63.37*	39.5±476.53*	38.5±10779.57	**<0.001**	19.0±4531.67	42.0±382.55	**<0.001**	16.0±28.69	38.0±74.96	**<0.001**	25.0±49.21	49.5±538.8	**<0.001**	38.0±15454.28	39.0±27.06	0.3528
AST on adm (U/L)	24.5[18.0-36.83]	22.0±29.04*	27.5±24.27*	27.5±19.37	**<0.001**	19.0±18.33	27.5±29.52	**<0.001**	18.0±18.65	26.0±33.7	**<0.001**	24.0±15.77	29.0±25.76	**<0.0005**	23.0±20.43	28.0±18.16	0.187
AST peak (U/L)	26.0[19.0-45.0]	23.0±39.2*	29.0±458.01*	41.0±723.15*	**<0.001**	20.5±306.36	29.0±366.48	**<0.001**	18.0±24.76	27.0±45.63	**<0.001**	26.0±31.19	32.0±518.64	**<0.0081**	45.0±1034.13	39.0±17.56	0.279
CK on adm (U/L)	106.0[66.0-171.25]	98.0±593.21*	121.0±779.65*	100.5±662.89	<0.0124	68.0±367.45	127.0±788.7	**<0.001**	60.0±95.28	124.5±744.67	**<0.001**	73.0±649.63	134.5±808.39	**<0.001**	99.0±75.4	102.0±919.9	0.4505
CK peak (U/L)	109.0[66.5-197.0]	99.0±624.95*	122.0±6413.72*	124.5±673.89	**<0.0265**	69.0±993.9	132.0±5169.41	**<0.001**	62.0±100.98	127.5±781.98	**<0.001**	73.0±1789.62	137.0±7157.57	**<0.001**	175.0±218.01	106.0±920.46	0.1499
**HAEMATOLOGIC ANALYSIS**																	
Platelet on adm x10^9 /L	224.0[180.25-272.0]	226.5±74.26	222.5±81.08	218.0±99.54	0.4348	244.0±79.88	216.0±77.15	**<0.001**	247.0±81.75	212.0±64.64	**<0.001**	247.0±67.27	216.0±84.33	**<0.0085**	212.0±88.36	225.0±104.99	0.1631
Platelet minx10^9 /L	224.0[178.0-272.0]	228.5±76.21	221.0±94.6	195.0±111.11	0.1046	240.0±83.84	216.0±87.62	**<0.0002**	244.0±84.7	217.0±67.09	**<0.0003**	236.0±69.03	215.0±100.66	**<0.0115**	170.0±97.77	223.0±115.31	0.087
**Leukocytes**																	
WBC on adm x10^9 /L	5.8[4.5-7.2]	5.8±2.15	5.8±2.86	5.8±2.08	0.4182	5.7±2.25	5.8±2.58	**<0.0134**	5.9±2.25	5.7±2.09	0.354	5.5±2.32	6.0±2.96	**<0.0045**	5.7±1.84	5.85±2.24	0.3985
Lowest WBC x10^9 /L	5.5[4.1-7.2]	5.65±2.25	5.4±11.14	4.9±1.86	0.2541	5.4±2.49	5.5±9.0	**<0.0279**	5.7±2.54	5.6±2.03	0.3527	5.1±2.45	5.4±12.61	**<0.0141**	4.9±1.93	5.05±1.78	0.3609
Lymphocytes on adm x10^9 /L	1.57[1.06-2.1]	1.8±0.76*	1.43±0.71*	1.16±0.83*	**<0.001**	1.6±0.8	1.5±0.74	0.0698	1.7±0.83	1.8±0.7	0.4752	1.57±0.7	1.39±0.71	0.1127	1.3±0.59	1.06±0.99	0.3599
Lymphocytes min x10^9 /L	1.49[0.89-2.08]	1.7±0.8*	1.34±0.78*	0.85±0.89*	**<0.001**	1.54±0.84	1.48±0.81	0.0817	1.67±0.85	1.72±0.76	0.3666	1.43±0.76	1.3±0.78	0.1295	0.83±0.61	0.88±1.07	0.403
**Red blood cells**																	
Hemoglobin on adm (g/l)	13.7[12.5-14.7]	13.75±1.62*	13.8±8.37	11.8±1.95*	**<0.001**	12.5±1.23	14.4±6.73	**<0.001**	12.6±1.14	14.6±1.31	**<0.001**	12.3±1.25	14.1±9.47	**<0.001**	11.2±1.42	12.0±2.29	0.2665
